# Supplementary material for: SARS-CoV-2-mediated dysregulation of metabolism and autophagy uncovers host-targeting antivirals
Source: Nat Commun. 2021 Jun 21;12:3818. doi: 10.1038/s41467-021-24007-w (PMC8217552; doi:10.1038/s41467-021-24007-w)
Supplement: Supplementary file 1 — Supplementary Information [file 41467_2021_24007_MOESM1_ESM.pdf]

Supplementary data (Figures and Tables)

"SARS-CoV-2-mediated dysregulation of metabolism and autophagy uncovers host-targeting antivirals" (Gassen et al.)

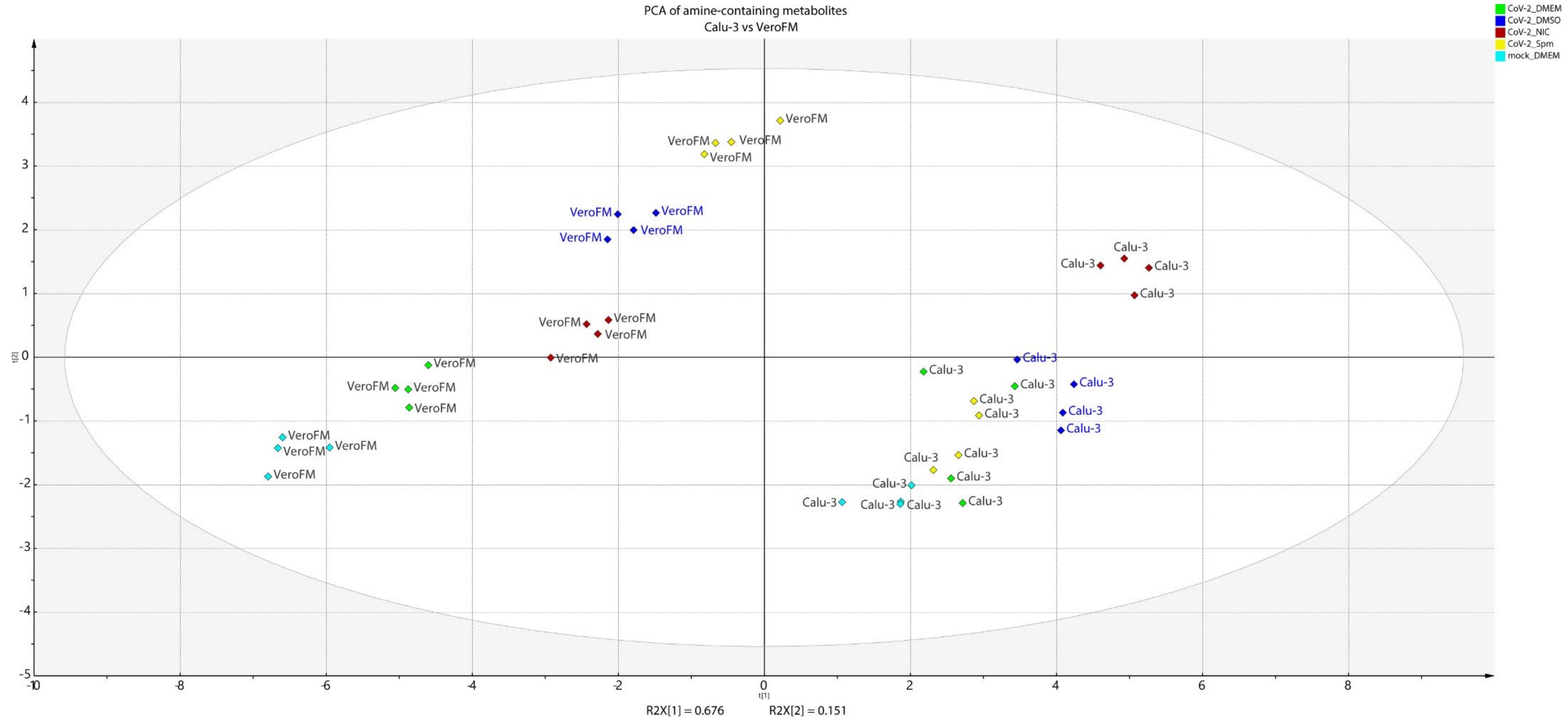

Supplementary Fig. 1. Principal component analysis (PCA) comparing VeroFM and Calu-3 cells in mock (mock_DMEM), SARS-CoV-2-infected (CoV-2 DMEM, 24 h p.i.) and SARS-CoV-2-infected and treated (with either drug vehicle (CoV-2 DMSO), niclosamide (CoV-2 NIC) or spermine (CoV-2 Spm). The variables used for this PCA analysis were all identified amine-containing metabolites (*n* = 4 per group, one experiment). NIC and Spm data refer to **Fig. 5**. For PCA analysis log10 transformed and pareto scaled and mean-centered peak areas of all samples were analyzed using SIMCA 13 (Umetrics).

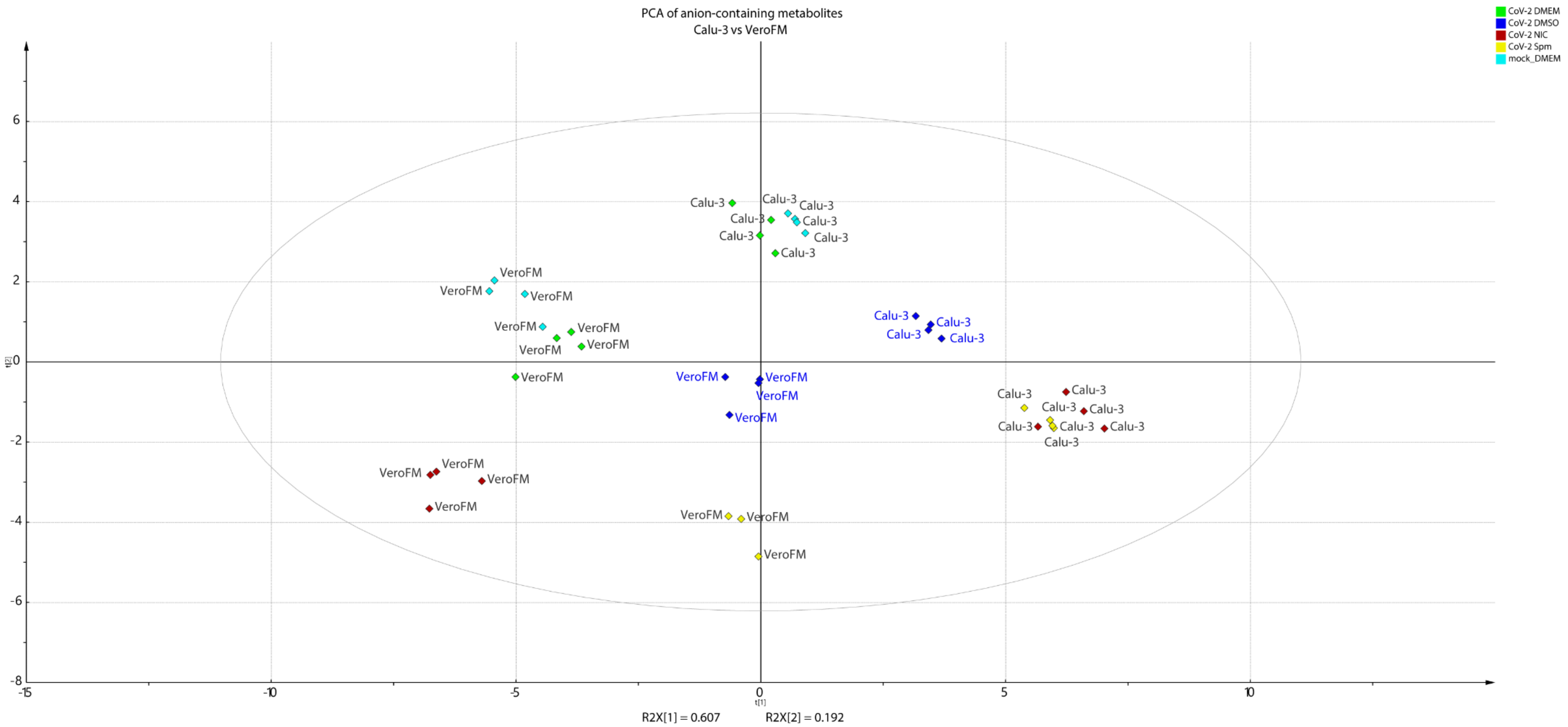

Supplementary Fig. 2. Principal component analysis (PCA) comparing VeroFM and Calu-3 cells in mock (mock_DMEM), SARS-CoV-2-infected (CoV-2 DMEM, 24 h p.i.) and SARS-CoV-2-infected and treated (with either drug vehicle (CoV-2 DMSO), niclosamide (CoV-2 NIC) or spermine (CoV-2 Spm). The variables used for the PCA analysis were all identified anion-containing metabolites ($n = 4$ per group, except $n = 3$ for VeroFM CoV-2 Spm, one experiment). NIC and Spm data refer to **Fig. 5**. For PCA analysis log10 transformed and pareto scaled and mean-centered peak areas of all samples were analyzed using SIMCA 13 (Umetrics).

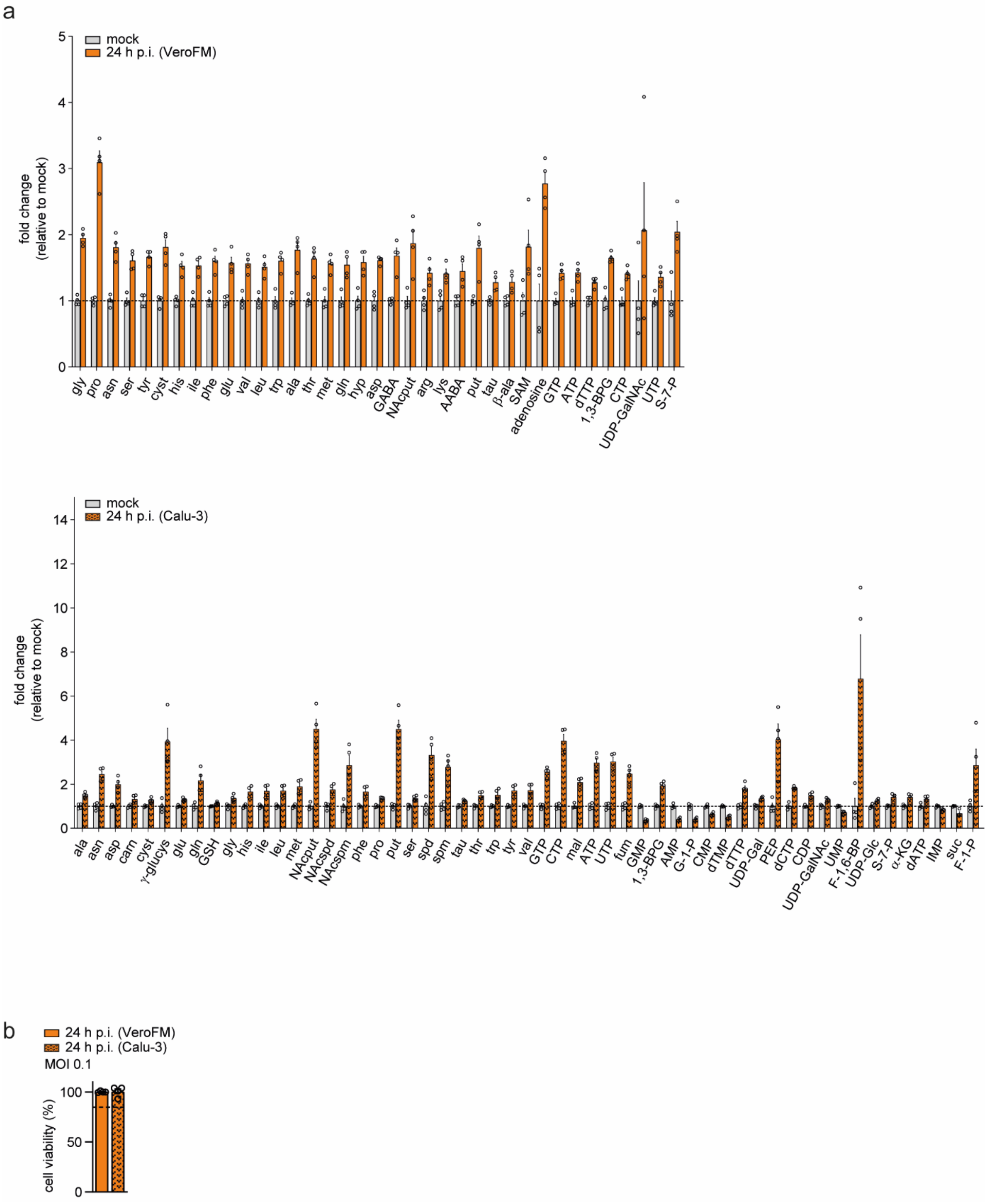

Supplementary Fig. 3. a Relative concentration of significantly (FDR-corrected p<0.05) changed host metabolites in VeroFM (37 metabolites) and Calu-3 (55 metabolites) cells 24 hours post SARS-CoV-2 infection (n = 4, mean with SEM, one experiment). All p-values were determined by a two-way ANOVA and Tukey´s post hoc test. P-values can be derived from **Supplementary Table 1 and 2. b** Cell viability assay (LDH-based) of supernatants from SARS-CoV-2-infected (MOI of 0.1) VeroFM and Calu-3 cells 24 h p.i. n = 4, mean with SEM, one experiment. Abbreviations: see **Supplementary Table 10**.

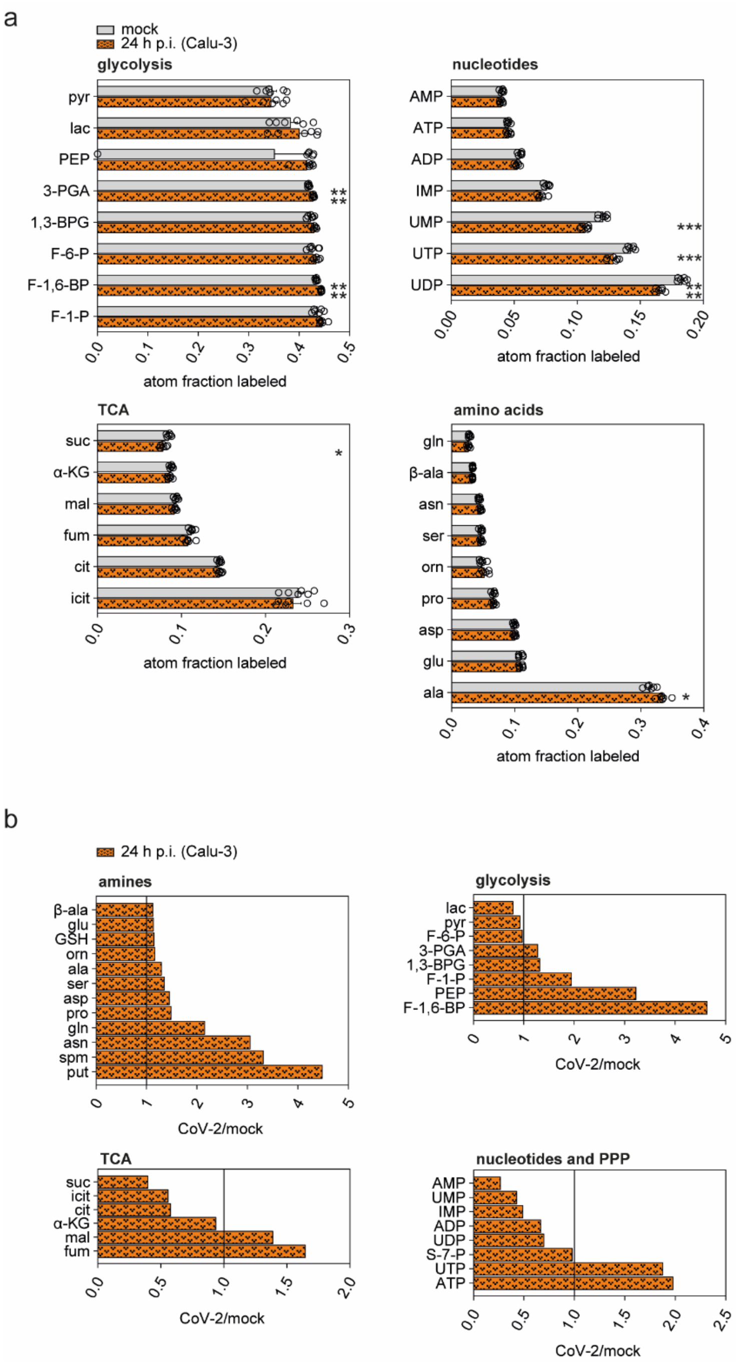

Supplementary Fig. 4. a ^{13}C isotope enrichment using U-$^{13}C_6$-labeled glucose as tracer in SARS-CoV-2-infected Calu-3 cells. Bar charts display the mean with SEM of the fraction of ^{13}C-labeled isotopes (atom fraction labeled in %) of each significantly altered metabolite analyzed in mock- and SARS-CoV-2-infected cells. Metabolites are assigned to their class or the metabolic pathway with which they are associated (glycolysis, TCA) or their metabolite class (nucleotides, amino acids). **b** Visualization of ratios of ^{13}C atom fraction labeling-corrected pool sizes of SARS-CoV-2-infected/mock-treated Calu-3 cells. Atom fraction corrected metabolite areas of the displayed metabolites were obtained by multiplying the cell-count-normalized metabolite areas with the atom fraction factor providing the ^{13}C isotope portion of each visualized compounds. Ratios >1 indicate increased isotope enrichment in the SARS-CoV-2-infected cells, while ratios <1 hint towards decreased isotope enrichment in SARS-CoV-2-infected cells. Metabolites are organized according to their metabolite class or pathway. For **a-b** n = 6 for mock and SARS-CoV-2-infected cells (one experiment). All p-values were determined by a two-way ANOVA and Tukey´s post hoc test with FDR correction. $p \leq 0.05$ (*), $p \leq 0.01$ (**), $p \leq 0.001$ (***), $p \leq 0.0001$ (****), $p > 0.05$ (not significant, ns). **Abbreviations:** 1,3-BPG, 1,3-bisphosphoglyceric acid; 3-PGA, 3-phosphoglyceric acid; α-KG, α-ketoglutarate; ADP, adenosine diphosphate; ala, alanine; AMP, adenosine monophosphate; asn, asparagine; asp, asparagine; ATP, adenosine triphosphate; cit, citric acid; F-1-P, fructose 1-phosphate; F-6-P, fructose 6-phosphate; F-1,6-BP, fructose 1,6-bisphosphate; fum, fumarate; gln, glutamine; glu, glutamic acid; GSH, glutathione (reduced); icit, isocitric acid; IMP, inosine monophosphate; lac, lactic acid; mal, malic acid; orn, ornithine; PEP, phosphoenolpyruvic acid; pro, proline; put, putrescine; pyr, pyruvic acid; S-7-P, sedoheptulose-7-phosphate; ser, serine; spm, spermine; suc, succinate; UDP, uridine diphosphate; UMP, uridine monophosphate; UTP , uridine triphosphate.

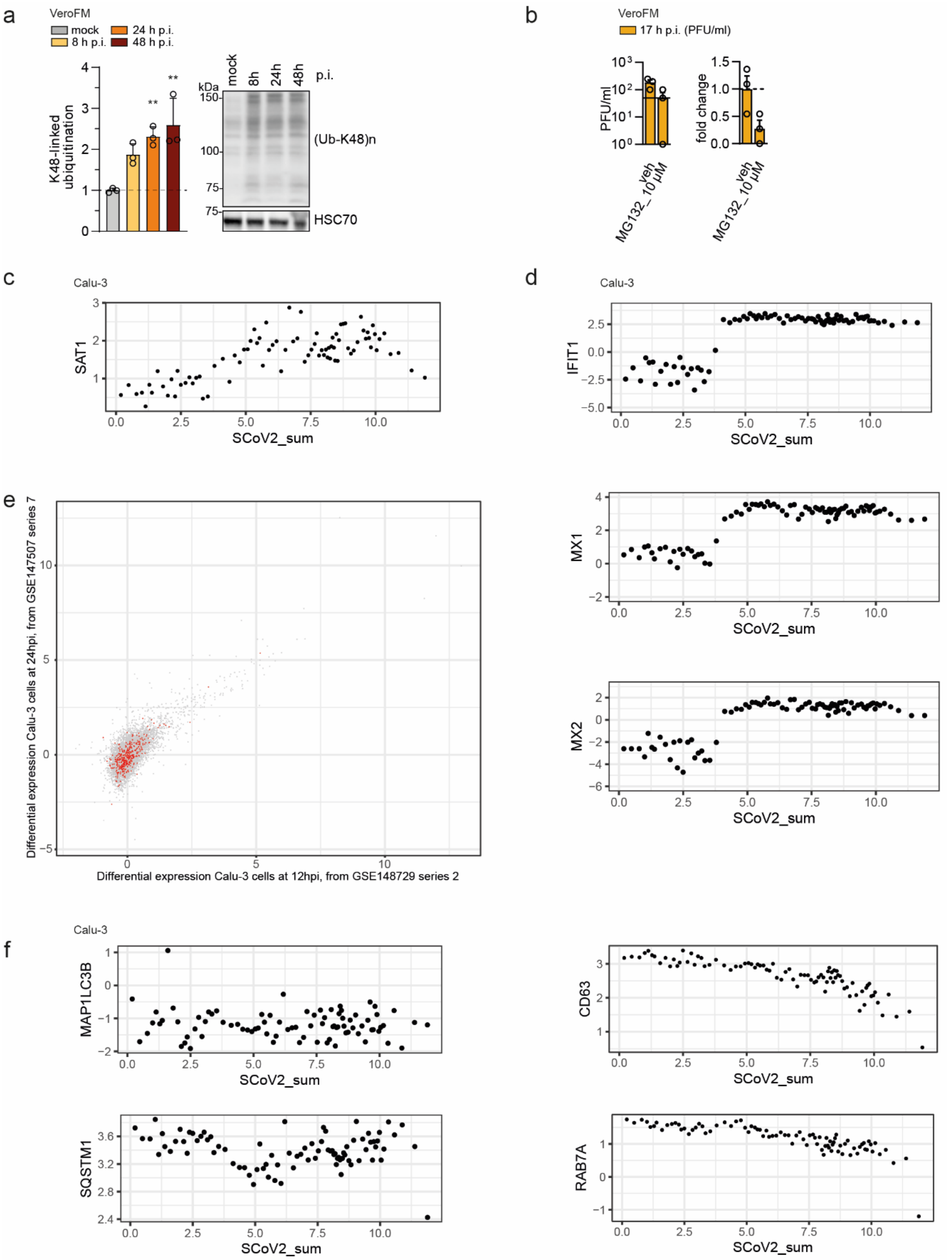

Supplementary Fig. 5. a Western blot analysis of K48-ubiquitinated proteins after SARS-CoV-2 infection in VeroFM cells; HSC70 served as a loading control. $n = 3$, mean with SEM, one experiment. P-values were determined by one-way ANOVA, Bonferroni post hoc. **b** SARS-CoV-2 propagation (PFU/ml) in infected VeroFM cells (MOI 0.0005) in response to 10 µM MG132 treatment (5 hours) or vehicle (DMSO) control 17 hours post infection. $n = 3$, mean with SEM, one experiment, Student t test p=0.3. **c-d** Comparison of *SAT1*, *IFIT1*, *MX1*, and *MX2* transcript levels with SARS-CoV-2 signatures in Calu-3 cells (SCoV2_sum). **e** The mRNA expression levels of TFEB-regulated genes are downregulated in SARS-CoV-2-infected Calu-3 cells. Differential expression values were calculated for SARS-CoV-2-infected Calu-3 cells compared to mock-treated cells using published RNA-sequencing datasets (GSE148729 and GSE147507). Shown are differential expression values for all genes (gray) and 399 TFEB target genes (red) **f** Comparison of *MAP1LC3B, CD63, SQSTM1,* and *RAB7A* transcript levels with SARS-CoV-2 signatures (SCoV2_sum). $p \leq 0.05$ (*), $p \leq 0.01$ (**), $p \leq 0.001$ (***), $p \leq 0.0001$ (****), $p > 0.05$ (not significant, ns).

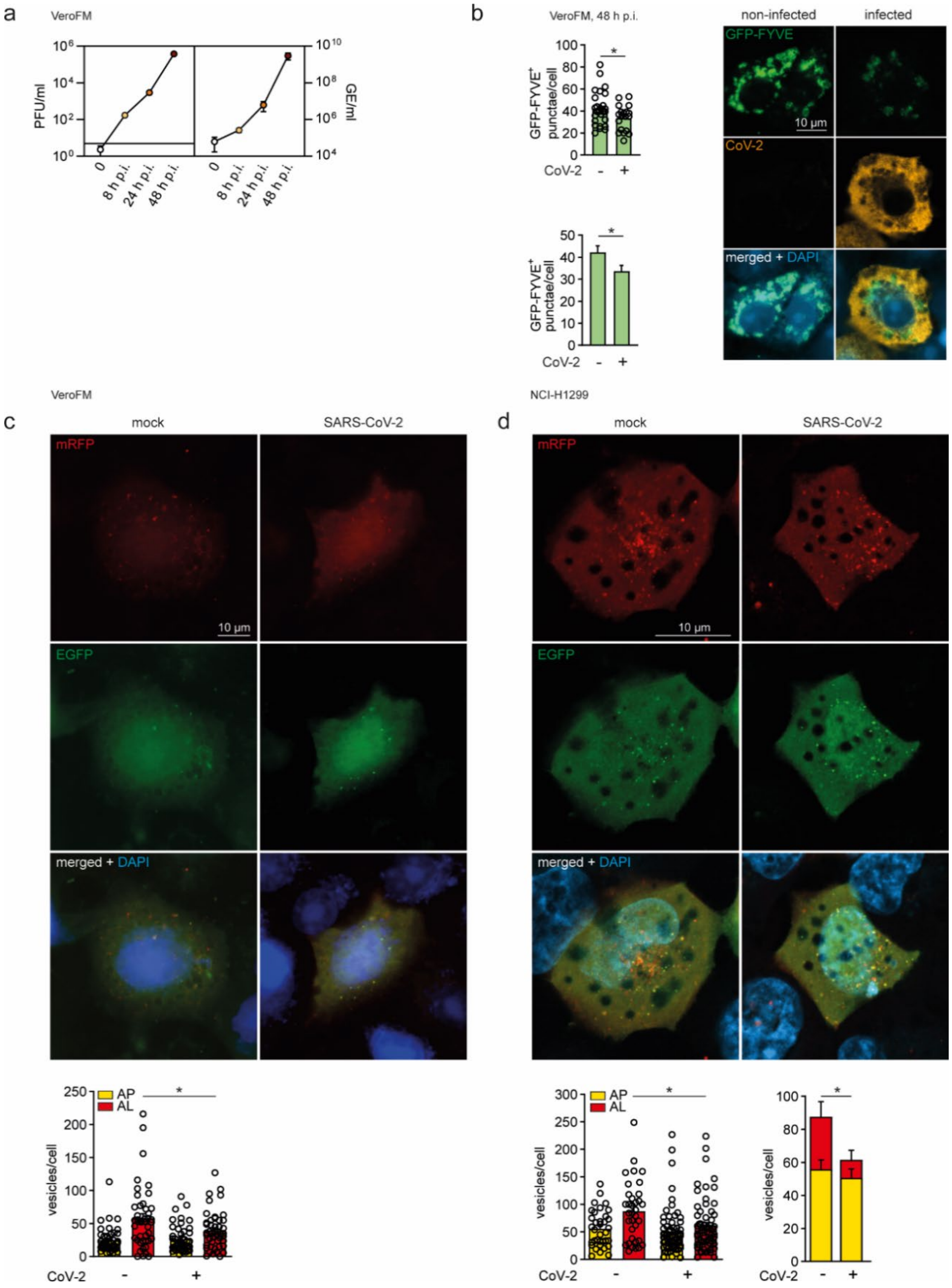

Supplementary Fig. 6. a Growth kinetics of SARS-CoV-2 in VeroFM cells. *n* = 3, mean with SEM, one experiment. **b** Quantification of GFP-FYVE punctae in SARS-CoV-2-infected and uninfected VeroFM cells; micrographs representative of those used for quantification 48 hours post infection. *n* = 27 cells (-CoV-2), *n* = 19 cells (+CoV-2), unpaired t test, mean with SEM. **c-d** Quantification of autophagolysosomes (ALs, defined as mRFP$^+$ EGFP$^-$ punctae) and autophagosomes (APs, defined as mRFP$^+$ EGFP$^+$ punctae) using tandem fluorescent-tagged LC3 in mock- and SARS-CoV-2-infected VeroFM (**c**: *n* = 44 cells (CoV-2; mock), *n* = 46 cells (CoV-2), two-way ANOVA, Tukey´s post hoc test, mean with SEM) and NCI-H1299 cells (**d:** *n* = 35 cells (CoV-2; mock), *n* = 64 cells (CoV-2), two-way ANOVA, Tukey´s post hoc test, mean with SEM); micrographs representative of those used for quantification. Lower right image in **c** is also shown in **Fig. 2b** in higher magnification. Vesicle/cell graph in **c** is shown without individual data points in **Fig. 2b**. $p \leq 0.05$ (*), $p \leq 0.01$ (**), $p \leq 0.001$ (***), $p \leq 0.0001$ (****), $p > 0.05$ (not significant, ns).

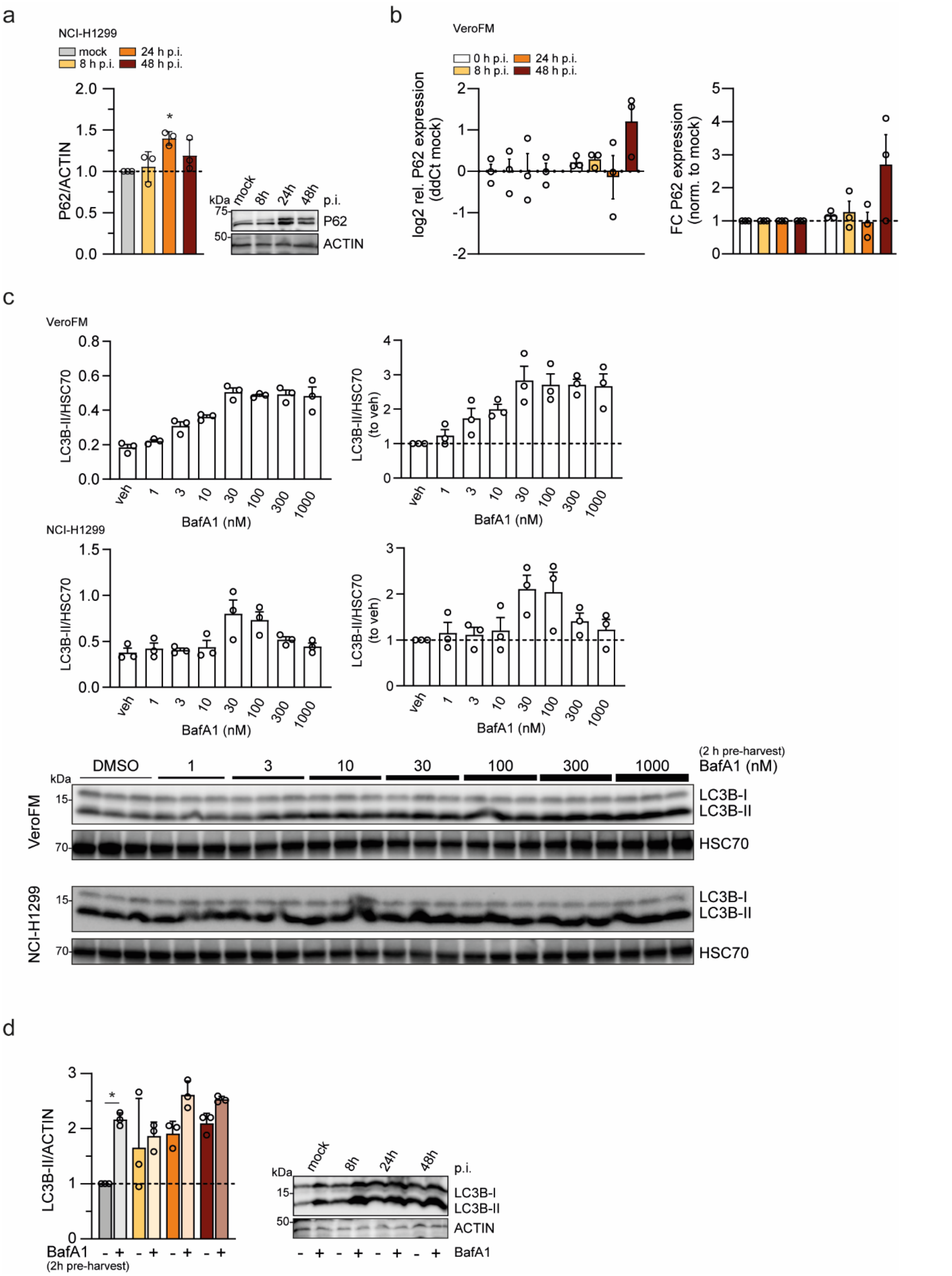

Supplementary Fig. 7. a Accumulation of autophagy marker P62 in SARS-CoV-2-infected NCI-H1299 cells. P-values were determined by one-way ANOVA, Bonferroni post hoc. **b** P62/*SQSTM1* mRNA transcript expression in uninfected (left, ddCt) or SARS-CoV-2 infected (right, ddCt normalized to mock) VeroFM cells at 8, 24, and 48 hours post infection. **c** Western blot analysis of LC3B-II levels relative to HSC70 loading control, in response to increasing concentrations of BafA1 (1-1000 nM) in VeroFM and NCI-H1299 cells for 2 hours. **d** Elevated LC3B-II levels in SARS-CoV-2-infected and bafilomycin A1 (BafA1)-pretreated NCI-H1299 cells indicate virus-induced autophagic flux inhibition. In all panels, error bars denote SEM derived from $n = 3$ biologically independent samples from one experiment. P-values were determined by two-way ANOVA, Sidak post hoc. $p \leq 0.05$ (*), $p \leq 0.01$ (**), $p \leq 0.001$ (***), $p \leq 0.0001$ (****), $p > 0.05$ (not significant, ns).

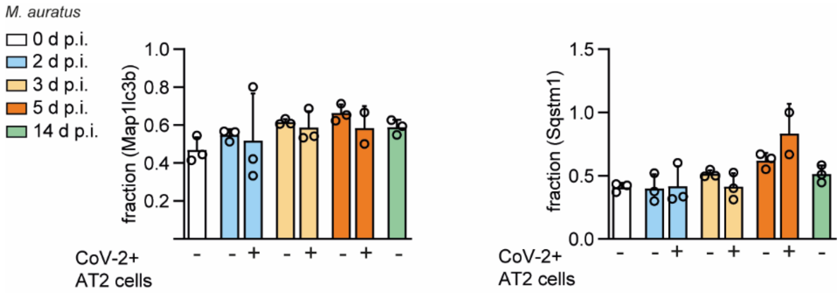

Supplementary Fig. 8. *Map1lc3b* and *Sqstm1* transcript levels in SARS-CoV-2-infected hamsters. Homogenized lung tissues of hamsters (*M. auratus*) were applied to single cell sequencing analysis at 0, 2, 3, 5, and 14 days post infection with SARS-CoV-2. Comparable *Map1lc3b* and *Sqstm1* transcript levels were identified in SARS-CoV-2-infected vs non-infected hamster lung AT2 cells. Of note, at time points 0 and 14 days post infection only uninfected AT2 cells were detected precluding a comparison. The published RNA-sequencing datasets can be found at GSE162208. $n = 3$, mean with SD from one experiment. (Exception: $n = 2$ for *Map1lc3b* and *Sqstm1* at 5 d p.i.). Comparisons were tested by two-way ANOVA, Tukey´s post hoc test. None of the relevant comparisons (day 0 vs days 2-14; mock vs infected animals) were significant.

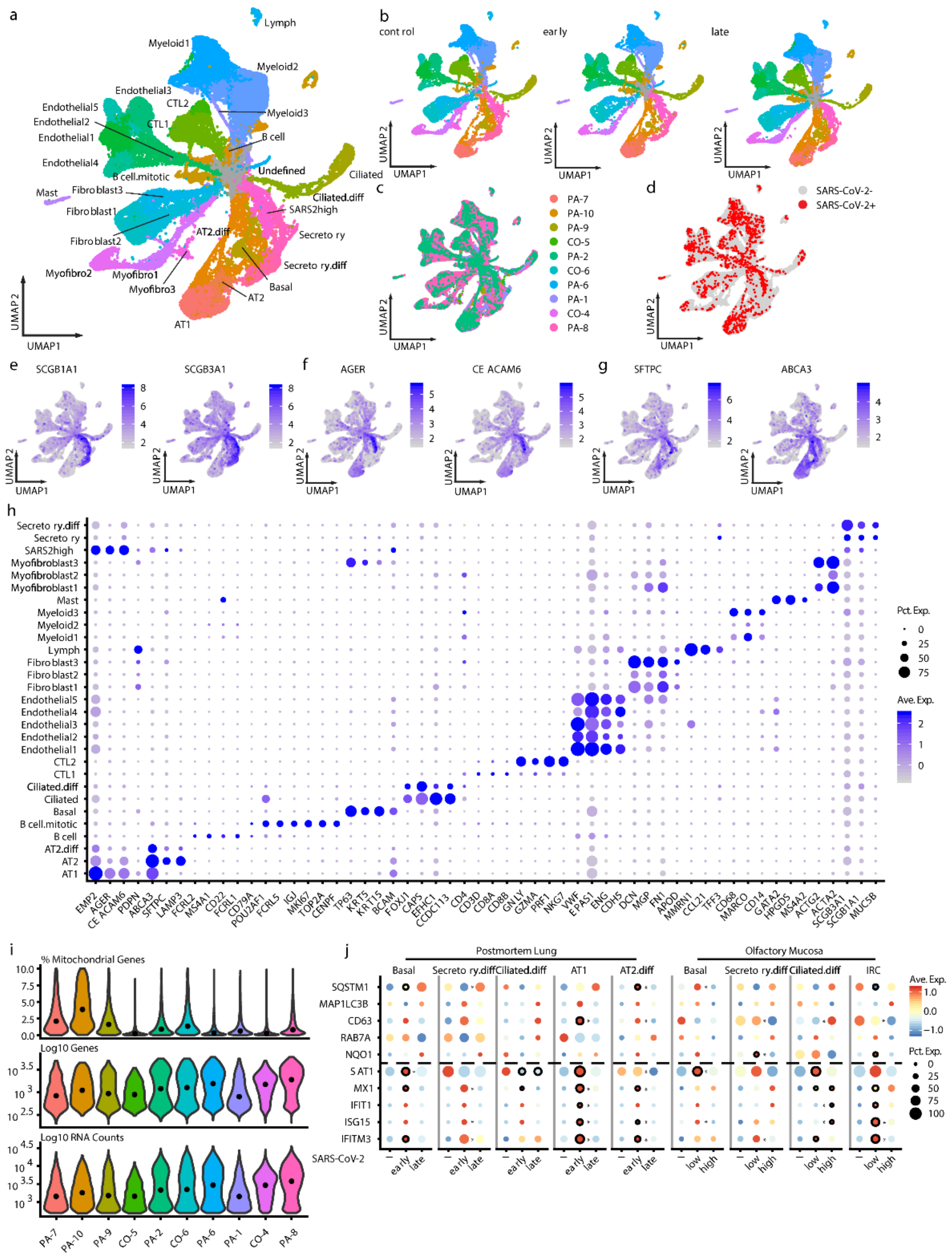

Supplementary Fig. 9. a Overview of cellular subpopulations inferred from single-nucleus sequencing (sNucSeq) and clustered by UMAP transcriptomic profiling of postmortem lung tissue and olfactory mucosa of SARS-CoV-2-negative controls and COVID-19 patients (n = 13). **b** when sorted according to SARS-CoV-2-negative controls (n = 6) and donors who deceased early (n = 3) or late (n = 4) in the course of COVID-19 treatment. **c** when colored by individual donor source; **d** when colored according to the presence or absence of SARS-CoV-2 RNA. **e** depicting relative *SCGB1A1* and *SCGB3A1* transcript levels as markers for secretory cells. **f** depicting relative *AGER* and *CEACAM6* transcript levels as markers for AT1 cells. **g** depicting relative *SFTPC* and *ABCA3* transcript levels as markers for AT2 cells. **h** Dot plot representing the average expression level (color) and percentage of cells (radius) expressing a selection of marker genes used to identify the different cell clusters. **i** Percentage of mitochondrial genes and the absolute quantity of host gene and RNA counts identified by sNucSeq, sorted by individual donors. **j** Dot plot representing the average expression level (color) and percentage of cells (radius) expressing a selection of autophagy-related transcripts within the previously defined cellular subtypes, and divided according to SARS-CoV-2-negative, early-, and late-deceased COVID-19 groups. Differential expression analyses was calculated with MAST, and p-values were adjusted with the Benjamini-Hochberg method. For comparisons to the uninfected control, significance is indicated by a black circle. For comparisons between conditions (early vs. late or low viral load vs. high viral load) significance is depicted by an asterisk. $p \leq 0.05$ (*). IRC – IFN Responsive Cell. Ave. Exp. = average expression, Pct. Exp. = percent of cells expressing the gene. The sequencing data are available under controlled access and require a Data Transfer Agreement in the European Genome-pheome Archive repository: (EGAS00001004689).

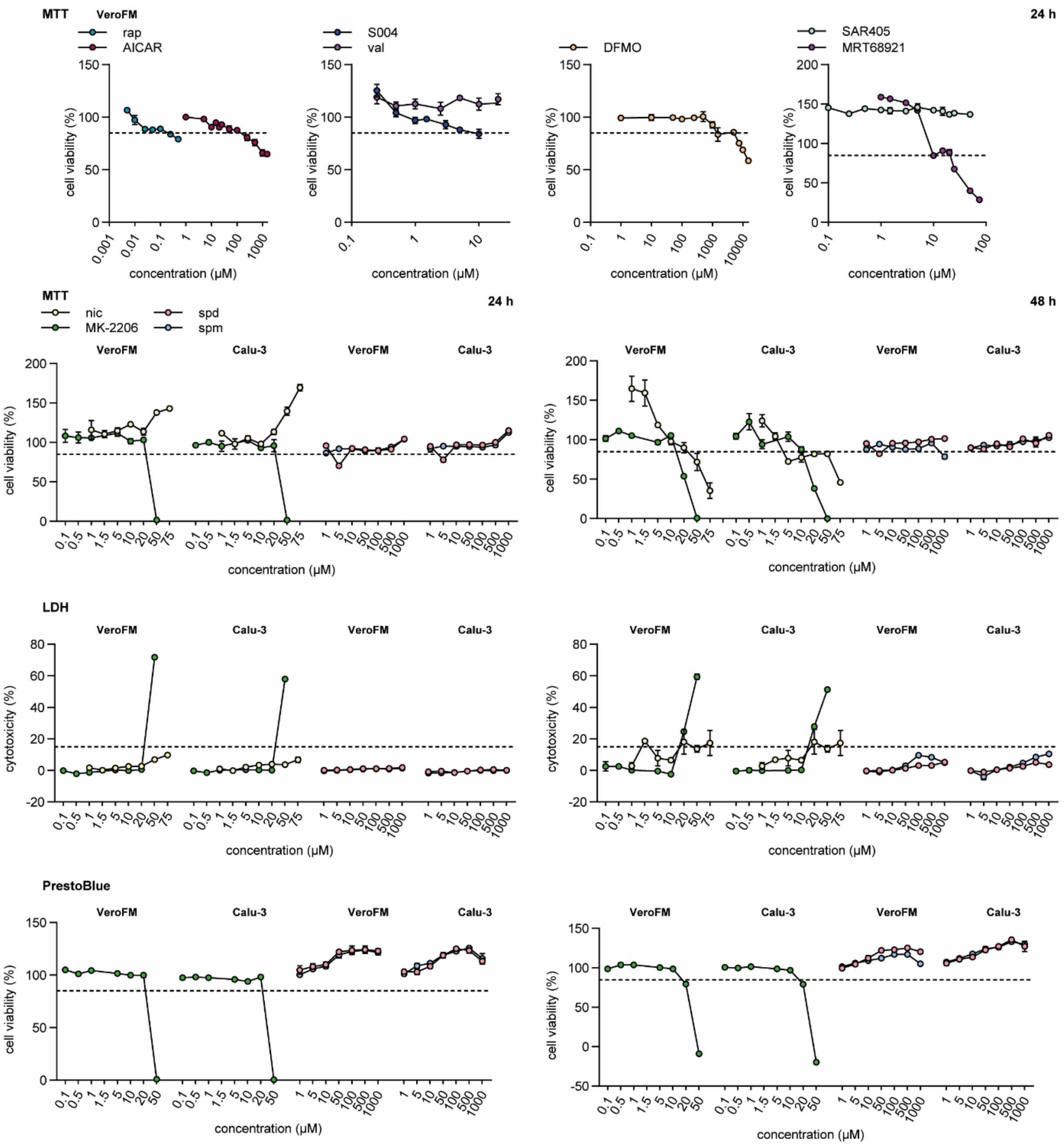

Supplementary Fig. 10. MTT cell viability assays performed on VeroFM cells in response to 24 hour-treatment with rap, AICAR, S004, val, DFMO, SAR405, and MRT68921. MTT cell viability, LDH cytotoxicity, and PrestoBlue cell viability assays performed on VeroFM and Calu-3 cells in response to 24- and 48-hour treatment with nic, MK-2206, spd, and spm. Of note, nic was incompatible with the PrestoBlue assay due to a change in the color of the solution and is not shown. *n* = 3 in general, *n* = 2 (MTT, spm 1000 µM, Calu-3, 24 h), *n* = 2 (MTT, nic, 75 µM, Calu-3, 48h), *n* = 2 (LDH, nic, 10 µM, Calu-3, 24 h), *n* = 2 (LDH, MK-2206, 20 µM, Calu-3, 24 h), mean with SEM. **Abbreviations:** AICAR, 5-Aminoimidazole-4-carboxamide ribonucleotide; DFMO, difluoromethylornithine; nic, niclosamide; rap, rapamycin; S004, SMIP-004; spd, spermidine; spm, spermine; val, valinomycin.

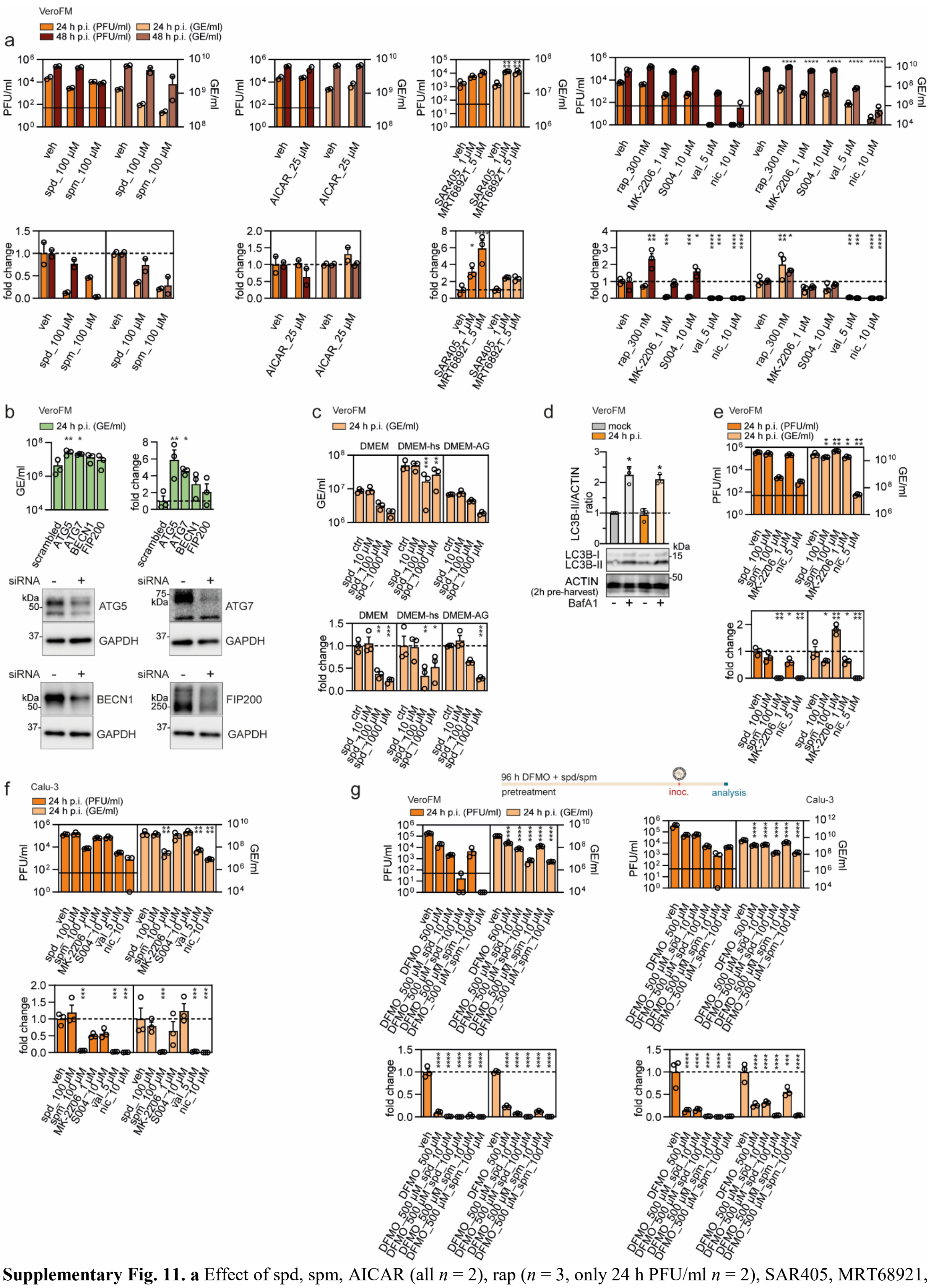

Supplementary Fig. 11. a Effect of spd, spm, AICAR (all *n* = 2), rap (*n* = 3, only 24 h PFU/ml *n* = 2), SAR405, MRT68921, MK-2206, S004, val, and nic (all *n* = 3) on SARS-CoV-2 replication in VeroFM cells. **b** Effect of siRNA knockdown of *ATG5*, *ATG7*, *BECN1*, and *FIP200* on SARS-CoV-2 propagation in VeroFM cells compared to a non-specific (scrambled) siRNA control (*n* = 3). Knockdown efficiency is shown by Western blot analysis. P-values were determined by one-way ANOVA, Dunnett's, mean with SEM. **c** Effect of growth medium composition on anti-SARS-CoV-2 activity of spd in VeroFM cells. DMEM was supplemented with either 10% FBS, 10% human serum (DMEM-hs), or 10% FBS and 1 mM aminoguanidine (DMEM-AG) to exclude unspecific ROS-induced SARS-CoV-2 inhibition. Upper panel GE/ml, lower panel fold-change. **d** Niclosamide-treated (10 µM) VeroFM cells were infected with SARS-CoV-2 (MOI = 0.0005) and incubated with bafilomycin

A1 (BafA1, 100 nM) or vehicle (DMSO) for 2 h before samples were harvested at 24 h post infection (h p.i.). The ratios of LC3B-II/actin were determined by Western blotting. ($n = 3$). P-values were determined by two-way ANOVA, Sidak post hoc. **e** Effect of 24-hour pre-treatment of spd, spm, MK-2206, and nic on SARS-CoV-2 propagation in VeroFM. **f** Effect of spd, spm, MK-2206, and nic on SARS-CoV-2 propagation in Calu-3 cells post infection, upper panel: PFU/ml, GE/ml, lower panel: fold change. **g** Effect of DFMO-mediated polyamine depletion (96 hours pretreatment) and subsequent DFMO/polyamine co-treatments on SARS-CoV-2 propagation in VeroFM and Calu-3 cells. Limit of detection for PFU/ml=50. **a-g** If not stated otherwise, p-values were determined from $n = 3$ (one experiment) by two-way ANOVA, Dunnett's, mean with SEM. **Abbreviations:** AICAR, 5-Aminoimidazole-4-carboxamide ribonucleotide; DFMO, difluoromethylornithine; nic, niclosamide; rap, rapamycin; S004, SMIP-004; spd, spermidine; spm, spermine; val, valinomycin. $p \leq 0.05$ (*), $p \leq 0.01$ (**), $p \leq 0.001$ (***), $p \leq 0.0001$ (****), $p > 0.05$ (not significant, ns).

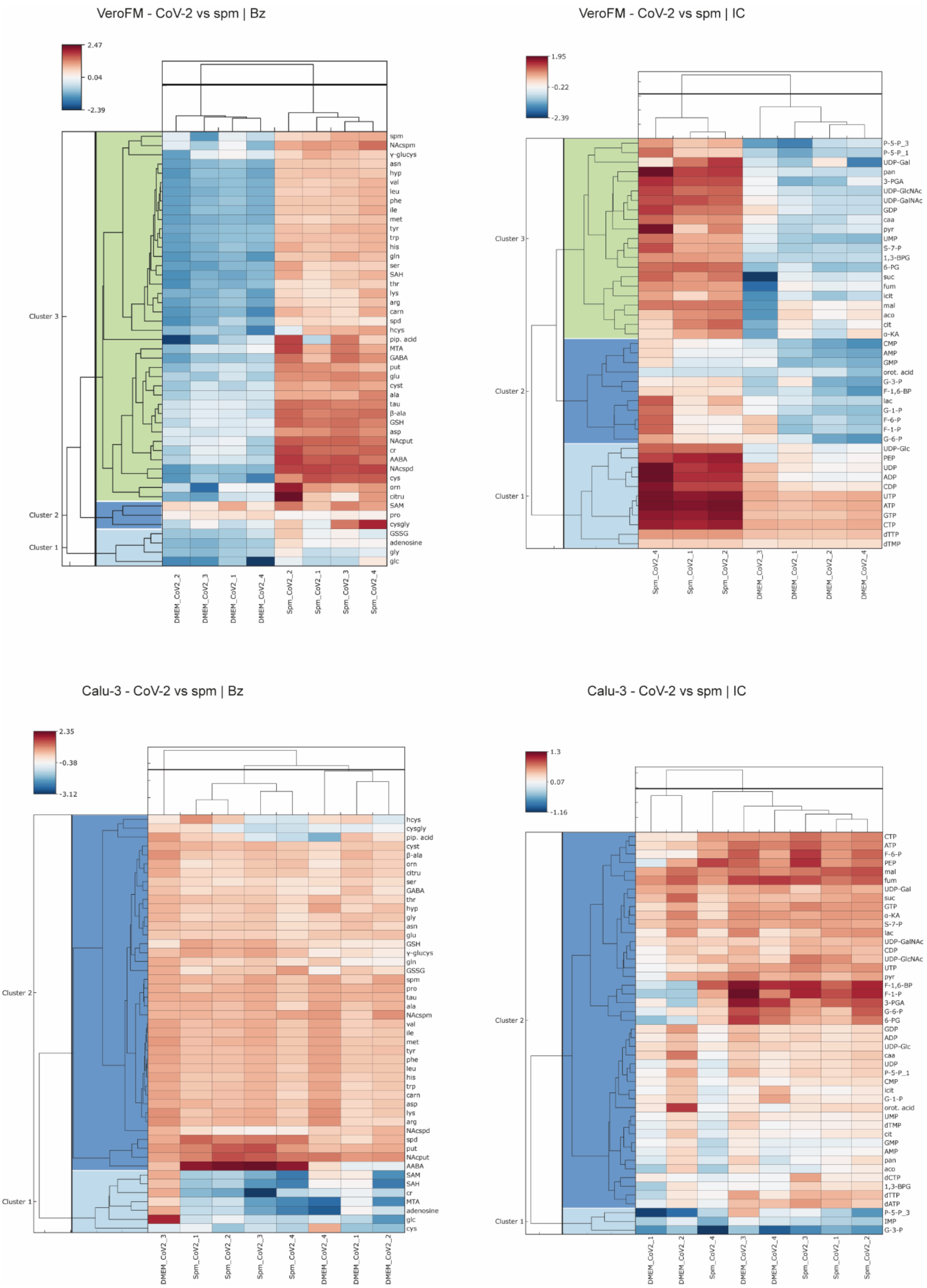

Supplementary Fig. 12. Heat maps of amine-containing (Bz) and anionic (IC) metabolites. Data visualizes the log10 transformed and z-score-scaled area values (see source data) of metabolites in VeroFM and Calu-3 cells treated with and without 100 µM spm at 24 hours post SARS-CoV-2 infection. A student t test was performed. $p \leq 0.05$ (*), $p \leq 0.01$ (**), $p \leq 0.001$ (***), $p \leq 0.0001$ (****), $p > 0.05$ (not significant, ns). Metabolite abbreviations are summarized in **Supplementary Table 10**.

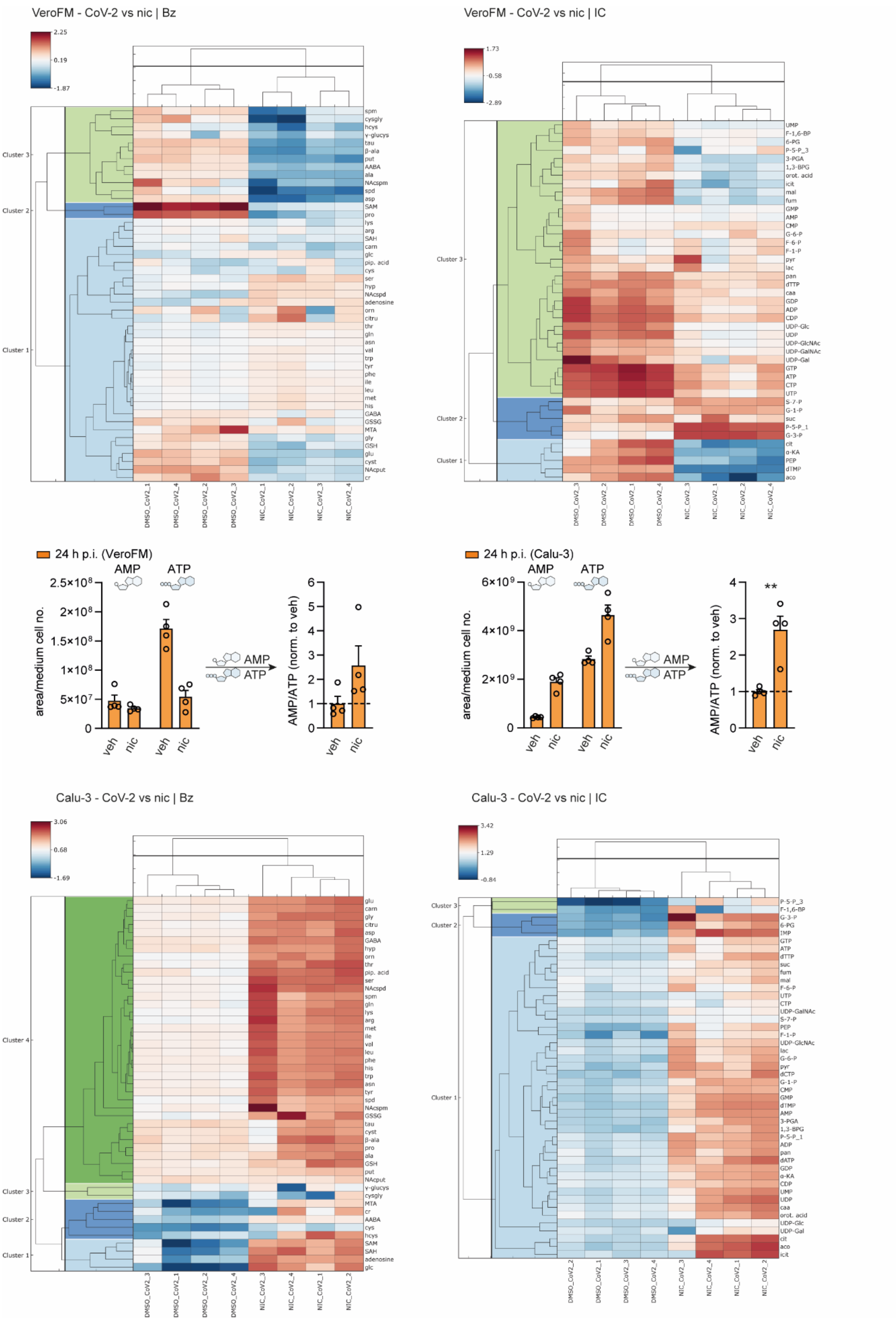

Supplementary Fig. 13. Heat maps of amine-containing (Bz) and anionic (IC) metabolites. Data visualizes the log10 transformed and z-score-scaled area values (see source data) of differentially regulated metabolites in VeroFM and Calu-3 cells treated with and without 10 µM NIC at 24 hours post SARS-CoV-2 infection. AMP and ATP raw data and AMP/ATP norm. to vehicle, $n = 4$, mean with SEM from one experiment. A Student´s t test was performed. $p \leq 0.05$ (*), $p \leq 0.01$ (**), $p \leq 0.001$ (***), $p \leq 0.0001$ (****), $p > 0.05$ (not significant, ns). Metabolite abbreviations are summarized in **Supplementary Table 10**.

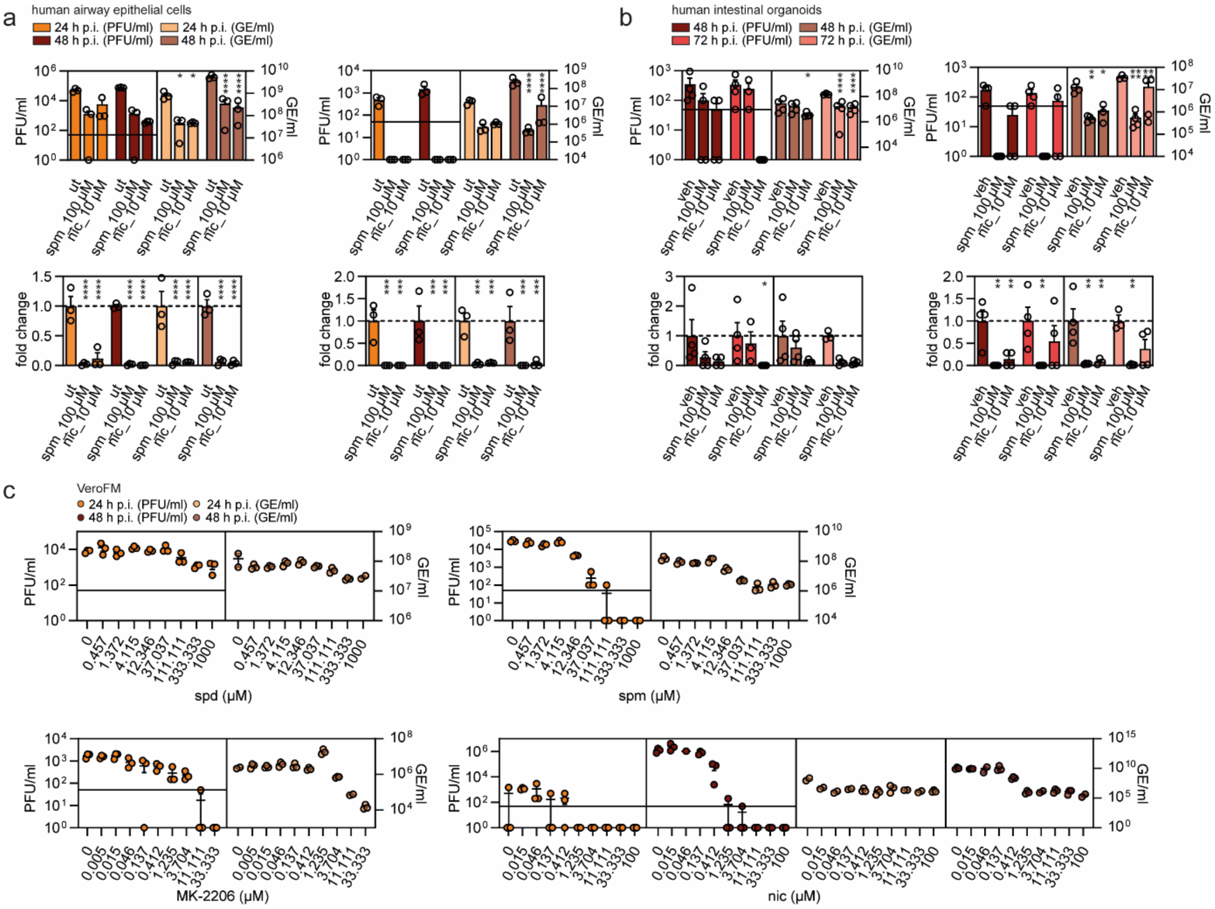

Supplementary Fig. 14. a-b The effects of spm and nic on SARS-CoV-2 propagation were examined in human airway epithelial cells at 24 and 48 hours post infection (**a**) and in human intestinal organoids at 48 and 72 hours post infection (**b**).
a Two independent experiments (twice, *n* =3, biologically independent samples). P-values were determined by two-way ANOVA, Dunnett's, mean with SEM. **b** Two independent experiments (twice *n* = 4, biologically independent samples), except *n* = 3 in experiment 1 for spm, 72 h p.i. (PFU/ml), veh, 72 h p.i. (GE/ml), and in experiment 2 for nic, 48 h p.i. (GE/ml), veh, 72 h p.i. (GE/ml). For both experiments p-values were determined by two-way ANOVA, Dunnett's, mean with SEM. **c** The putative antiviral effects of spd, spm, MK-2206, and nic were evaluated for dose-dependence (IC50 determination) at 24 (spd, spm, MK-2206) and 48 (nic) hours post SARS-CoV-2 infection. For MK-2206, *n* = 3 in general, *n* = 2 for 0 µM and 11.11 µM, 24 h p.i. (GE/ml). For nic, *n* =3 in general, *n* = 1 for 0.046µM, 48 h p.i. (PFU/ml), *n* = 2 for 0 µM, 0.015 µM, 0.046 µM, 0.137 µM, 11.11 µM, 33.33 µM, 24 h p.i. (GE/ml), *n* = 2 for 0.046 µM, 3.704 µM, and 100 µM, 48 h p.i. (GE/ml), mean with SEM. $p \leq 0.05$ (*), $p \leq 0.01$ (**), $p \leq 0.001$ (***), $p \leq 0.0001$ (****), $p > 0.05$ (not significant, ns). **Abbreviations:** nic, niclosamide; spd, spermidine; spm, spermine.

a

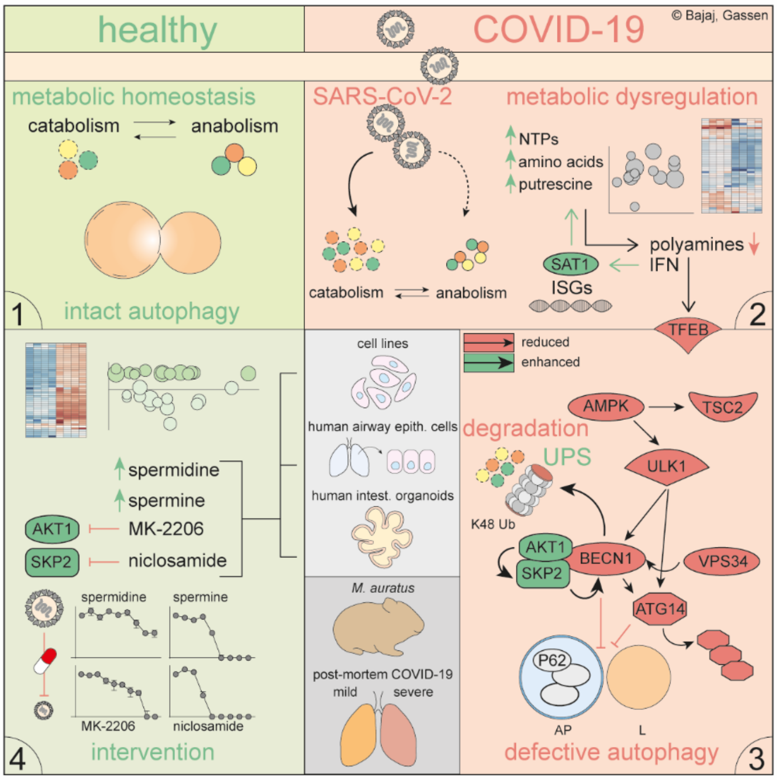

b

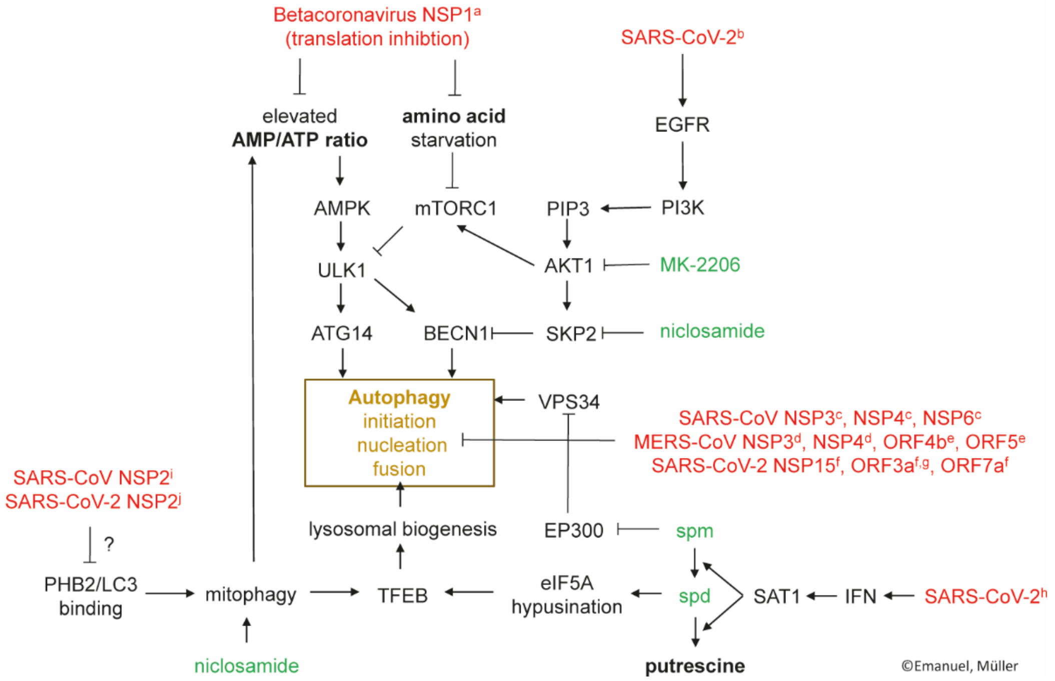

Supplementary Fig. 15. Graphical abstract and working model. **a** Graphical abstract summarizing the key findings of the study. **b** Working model including hypothesized interventions of SARS-CoV-2 based on previously published data (references for the red superscript letters a[1-4]; b[5]; c[6]; d[7]; e[8]; f[9]; g[10,11]; h[12]; i[13]; j[14]) and as mentioned in the discussion. Copyrights Thomas Bajaj, Nils C. Gassen (**a**), Jackson Emanuel, Marcel A. Müller (**b**).

Supplementary Table 1. VeroFM metabolomics

**Metabolite**	**SARS-CoV-2-infected**				**Mock-infected**				
	**area/medium cell number**				**area/medium cell number**				**Significance infected vs mock**
	**rep1**	**rep2**	**rep3**	**rep4**	**rep1**	**rep2**	**rep3**	**rep4**	***p***
**glycine**	54015.936	50755.126	49265.734	56250.224	25514.503	25543.971	27336.900	29493.889	<0.001
**proline**	29568740.660	24836329.850	30196937.490	32783021.590	8777666.466	9310508.168	9865928.424	9996847.510	0.001
**asparagine**	1229725.000	963400.290	1138249.095	1058883.362	665294.204	608088.882	542411.475	611553.567	0.002
**serine**	248277.373	209413.633	211261.036	242006.218	159663.299	135615.159	138172.298	133434.758	0.003
**tyrosine**	46529757.260	40606561.170	44357330.550	46557909.680	24469589.990	24437590.690	28963252.440	29043388.460	0.003
**cystathionine**	3796505.193	3395957.275	4349019.966	4443964.020	2254664.933	2304116.663	1935888.542	2330795.539	0.003
**histidine**	9904582.182	7898345.658	8732883.997	8945573.743	5858426.132	5325140.481	5906758.738	6178602.660	0.003
**isoleucine**	2158680.494	1778754.019	2121892.334	1961899.473	1211826.303	1209086.958	1329807.902	1476452.846	0.003
**phenylalanine**	3389062.089	2663183.856	3103336.989	3053249.384	1774319.870	1781449.502	1922351.260	2172022.541	0.003
**glutamic acid**	49800406.820	42681315.270	39094217.240	40404620.080	26108843.090	25070719.090	29321020.110	28913081.680	0.003
**valine**	1987813.977	1624318.556	1894863.681	1773790.781	1107810.534	1043054.124	1172519.611	1342146.085	0.003
**leucine**	2445007.311	1948584.693	2256546.944	2170977.601	1395448.575	1309207.032	1481112.193	1649346.315	0.004
**tryptophane**	5177756.994	4225546.393	4861490.099	4971552.267	2676212.639	2801510.916	2965012.146	3541223.250	0.004
**alanine**	1287237.788	1006030.603	1380928.411	1336452.566	695123.506	680769.617	660734.874	794546.816	0.004
**threonine**	585900.825	482492.609	618175.517	639326.290	340666.217	330367.134	358296.196	392248.304	0.004
**methionine**	1157779.614	945626.635	1077716.967	1063214.982	623901.138	614119.407	681192.301	803381.626	0.004
**glutamine**	24745893.660	19231155.440	23693255.930	20173448.170	13224708.150	13005389.210	14000271.270	16669745.760	0.006
**4-hydroxyproline**	545965.805	434304.613	547071.461	464049.099	277247.599	288678.113	316295.817	375650.422	0.006
**aspartic acid**	1322722.000	1343433.040	1234878.740	1328250.612	746107.225	703925.305	874494.228	920725.643	0.008
**gamma-aminobutyric acid**	34351.741	26925.571	34236.394	37964.693	18655.119	20598.669	19635.873	20419.496	0.009
**N-acetylputrescine**	1578653.212	1257200.492	1427573.412	921404.453	673269.203	623073.727	830639.625	646772.198	0.016
**arginine**	11460912.530	8627996.732	10335083.890	9295058.444	6564461.484	6004199.808	7313551.447	8099132.628	0.017
**lysine**	43712009.800	36742680.190	38226314.290	34669646.840	24472642.520	23431369.170	29642458.790	31299722.750	0.018
**alpha-aminobutyric acid**	10273307.360	7744041.766	10599927.140	8433369.707	5922563.933	6851092.110	5958511.572	6830068.718	0.019
**putrescine**	83617448.290	49815188.640	73460346.760	71750176.670	38968198.740	36789573.100	37822882.350	41530096.640	0.023
**taurine**	16370933.310	13260434.770	15583575.520	13426902.580	10802540.320	11486599.060	11469574.110	12134095.280	0.023
**beta-alanine**	6146841.494	5290365.776	6436089.054	5006980.126	4212378.285	4694466.519	4125540.489	4787342.177	0.029
**S-adenosylmethionine**	423471.628	307152.537	235546.967	247455.532	132196.411	179803.304	137255.517	219879.138	0.029

**adenosine**	171546.442	148593.042	139123.182	182976.454	34318.461	86217.499	30990.739	80231.851	0.037
**GTP**	9464503.124	9593431.636	10374852.070	11036570.850	6755498.695	6915018.305	6876696.692	7923845.437	0.021
**ATP**	80304826.280	86086743.780	91683955.280	97671778.040	58817906.310	60924818.910	57660849.340	72008302.180	0.027
**dTTP**	737720.833	808159.855	839124.791	841528.510	662452.405	610937.570	585139.668	661744.893	0.027
**1,3-bisphosphoglyceric acid**	6843712.798	6572650.522	6626495.003	7289922.458	3634725.624	4252337.294	3721529.116	4984876.388	0.027
**CTP**	8105046.455	8324426.142	8677184.628	9410438.474	5778829.132	5796428.869	5707661.658	7218511.865	0.027
**UDP-GalNAc**	9377.997	27939.311	14115.715	5029.836	4931.339	3474.989	12871.119	6074.039	0.027
**UTP**	36966556.680	39173827.850	43423994.910	46003711.810	29082298.580	29328863.600	28260072.960	34999751.550	0.027
**sedoheptulose-7-phosphate**	388512.905	442910.059	558541.728	432498.087	174051.519	187255.467	211140.607	320299.873	0.034
									significant (p<0.05)

Supplementary Table 2. Calu-3 metabolomics

Metabolite	SARS-CoV-2-infected				Mock-infected				
	area/medium cell number				area/medium cell number				Significance infected vs mock
	rep1	rep2	rep3	rep4	rep1	rep2	rep3	rep4	*p*
**alanine**	7095112.389	7947875.357	7998062.632	8882842.299	4939173.767	5847966.866	5831442.859	4781432.951	0.007
**asparagine**	5760524.917	4774819.653	5804383.575	4547269.398	2058096.960	2453889.392	2304371.498	1708012.506	0.002
**aspartic acid**	1906305.610	2198870.885	2523197.717	2840966.185	1175753.600	1169475.004	1286751.556	1135861.400	0.010
**carnosine**	1812582.506	1846997.587	2383187.254	2351367.038	1522989.863	1643008.655	1733675.291	1466753.889	0.046
**cystathionine**	103726252.000	104256550.200	119243044.800	93216215.330	78776160.850	85544019.730	86912786.170	82717034.240	0.025
**gamma-glutamylcysteine**	288325.798	219075.301	408765.062	231603.438	53430.440	63964.717	99572.798	74513.959	0.003
**glutamic acid**	202085002.200	220685520.500	193730769.200	203023606.000	155783466.900	156911943.900	166877093.600	148546529.400	0.003
**glutamine**	128535926.600	127460717.400	208065948.800	177174617.500	69928473.450	76986919.490	87141131.180	62147484.290	0.010
**glutathione_red (GSH)**	612043997.000	622347961.000	640703068.700	683781944.800	562098577.100	562252384.400	548067603.900	556655473.100	0.017
**glycine**	704363.007	667479.486	799679.169	597494.845	524544.885	520150.628	555856.347	436534.189	0.017
**histidine**	68900303.790	71436238.650	94189900.640	89651551.030	50085364.180	47430148.270	53793568.960	44872709.340	0.010
**isoleucine**	20273494.540	21845415.980	28479484.480	28064842.470	14369779.190	14942202.990	15887922.630	13243710.650	0.011
**leucine**	19642732.640	20051804.150	25920848.250	26218356.160	13144491.070	13745561.570	14713049.960	12348482.570	0.010
**methionine**	8289383.717	8571288.901	11634273.760	11377025.430	5147484.745	5369388.885	5814432.136	4817653.838	0.010
**N-acetylputrescine**	402748.542	479727.155	374901.494	576718.661	101987.753	95862.394	122490.940	87164.863	0.001
**N-acetylspermidine**	5411396.789	6705320.581	7053960.947	5400943.376	3800794.877	3492423.798	3910959.541	2806066.233	0.007
**N-acetylspermine**	335127.826	735187.700	468421.298	663254.035	256937.902	171544.949	196319.370	148416.588	0.011
**phenylalanine**	25428029.200	25876647.320	34199473.650	33673422.590	17592699.030	18214695.450	19344505.280	16784153.220	0.011
**proline**	335345426.900	370687840.900	373887730.700	366019388.100	284713665.900	270265370.100	259716237.400	253138005.300	0.002
**putrescine**	57343144.890	65384611.360	78485695.720	51109185.180	14938951.030	13877811.960	15561886.290	11813645.580	0.001
**serine**	1101377.008	955408.176	1134650.388	958167.249	803235.171	755459.174	820329.113	672119.289	0.010
**spermidine**	49723440.830	83957746.340	90413903.320	68962582.410	32153434.710	18262496.540	23539432.810	14438312.540	0.007
**spermine**	33090684.940	56197581.400	48130740.050	52118600.180	20422701.800	14809058.990	18756810.020	14188446.020	0.004
**taurine**	25416980.540	26261989.670	28382675.590	27537795.330	20998098.200	22638155.460	22914305.260	20332471.190	0.007
**threonine**	4136189.555	3879772.924	4960696.071	5006506.147	3132689.140	3224526.826	3055486.284	2693495.115	0.010
**tryptophan**	34527077.920	39650844.840	49855351.860	53089893.460	28790012.150	29924044.050	31058577.420	27910660.840	0.045
**tyrosine**	350029901.500	380202635.100	469693383.400	483082620.000	251334721.400	239288769.900	261255153.000	233159807.400	0.011
**valine**	13104556.890	14790965.850	18877641.860	18780594.650	9591562.953	9617156.217	10377783.350	8522680.385	0.011

**GTP**	200393415.700	228616439.800	243790388.100	228884986.800	86906995.090	95949493.760	93455231.260	76168561.250	<0.001
**CTP**	96996328.420	96035205.790	125550781.100	125050570.200	24525098.620	31613331.090	30465756.660	25386275.430	<0.001
**malic acid**	1616295668.000	1973177863.000	1945222444.000	1749568180.000	831622272.800	1008114646.000	817751369.300	831931942.700	<0.001
**ATP**	1997122933.000	2113913941.000	2667938549.000	2491548444.000	717488379.800	836738290.300	897437453.400	673202386.700	<0.001
**UTP**	567687644.500	621606891.700	750553389.200	742866170.400	205941266.200	250714198.500	245868296.600	184978634.700	<0.001
**fumaric acid**	137535199.800	169657490.800	182625138.100	196201617.500	63627484.730	75769227.090	79680896.060	58803317.590	0.001
**GMP**	44022773.070	55280215.780	49796249.830	41435877.150	133380487.500	124526507.800	127660754.700	138879531.800	0.001
**glyceric acid 1,3-biphosphate**	37988026.310	49412111.320	47012362.590	45368439.520	20627180.040	23847427.180	23179263.930	25233712.140	0.001
**AMP**	236727682.700	334738399.700	289772372.000	233741454.300	670086479.800	651023332.600	620233117.800	771033177.300	0.002
**glucose-1-phosphate**	9853200.032	12480244.200	10005035.230	14308815.770	24875962.870	29995410.460	28657643.140	31953816.210	0.002
**CMP**	19590980.800	23812247.020	20961396.100	18677886.640	32369843.800	30280942.430	31207347.300	35240411.540	0.003
**dTMP**	3574361.000	4502666.563	4766748.392	3726828.833	8044370.458	7965694.640	8403916.409	7961896.699	0.006
**dTTP**	9901428.320	12201276.320	14644664.050	12288954.720	6554699.485	7220923.129	7567557.785	6185052.283	0.006
**UDP-galactose**	1316529.625	1450396.129	1364678.549	1511926.815	1060402.939	1079606.463	1115136.620	918183.760	0.006
**phosphoenolpyruvic acid**	22973934.760	44095027.340	59710902.260	48145909.730	10822975.650	8690143.837	15315898.690	8580809.459	0.007
**dCTP**	931579.860	875701.344	875898.740	872461.450	466095.098	484618.953	578003.726	408609.556	0.007
**CDP**	22775054.990	27848023.550	29310978.810	25676250.810	17375364.590	19332756.830	17394059.470	17696443.550	0.007
**UDP-N-acetylgalactosamine**	24031029.800	27568717.820	28726298.340	26528588.780	19011261.220	21591643.660	22239155.840	20212590.420	0.007
**UMP**	380326695.600	471363943.800	476155302.700	425838004.300	617855033.700	593599551.800	634996512.100	640323540.500	0.010
**fructose 1,6-bisphosphate**	121126572.200	102211850.500	362970488.200	315690493.400	24971575.530	24136658.090	68155591.650	15707536.130	0.012
**UDP-glucose**	7017249.486	8275794.148	7869139.169	7529624.874	6899664.397	5935308.892	6129666.711	5653994.891	0.016
**sedoheptulose-7-phosphate**	21737975.480	27664487.190	29033048.080	27127584.350	19198279.130	17202179.340	19476854.370	18628226.990	0.016
**alpha-ketoglutaric acid**	735051692.900	995236129.700	966501251.700	917112612.700	630062545.000	659621006.900	695809623.900	578625483.500	0.017
**dATP**	1096058.980	1294664.305	1445223.890	1441213.302	988565.567	954501.929	1156744.541	881674.527	0.035
**IMP**	1780866.268	2067965.224	2207376.978	2004470.828	2421055.274	2253854.381	2446194.703	2517275.877	0.035
**succinic acid**	824615717.000	1231535537.000	840421863.000	824664331.200	1392852818.000	1403288614.000	1386748025.000	1331161449.000	0.043
**fructose-1-phosphate**	10490396.460	10405260.210	29618620.520	20054823.950	6603416.526	5616544.379	7826350.388	4711544.440	0.043
									significant (p<0.05)

Supplementary Table 3. Atom fraction labeling (AFL) data of amine (Bz)- and anion (IC)-containing metabolites of the ^{13}C isotope enrichment analysis using U-$^{13}C_6$-labeled glucose as tracer in SARS-CoV-2-infected Calu-3 cells. Metabolite abbreviations are summarized in **Supplementary Table 10**.

**Bz**														
	**BIO_SAMPLE_ID**	115	116	117	118	119	120	109	110	111	112	113	114	
	**ORGANISM**	*H. sapiens*	*H. sapiens*	*H. sapiens*	*H. sapiens*	*H. sapiens*	*H. sapiens*	*H. sapiens*	*H. sapiens*	*H. sapiens*	*H. sapiens*	*H. sapiens*	*H. sapiens*	
	**TISSUE**	Calu-3	Calu-3	Calu-3	Calu-3	Calu-3	Calu-3	Calu-3	Calu-3	Calu-3	Calu-3	Calu-3	Calu-3	Calu-3
	**TREATMENT**	mock	mock	mock	mock	mock	mock	CoV-2	CoV-2	CoV-2	CoV-2	CoV-2	CoV-2	mock vs CoV-2
	**TRACE**	13C glc	13C glc	13C glc	13C glc	13C glc	13C glc	13C glc	13C glc	13C glc	13C glc	13C glc	13C glc	p-values
	**average cell number (xE6)**	1.33	1.33	1.33	1.33	1.33	1.33	1	1	1	1	1	1	
	**ala**	0.311	0.317	0.326	0.321	0.303	0.313	0.323	0.332	0.350	0.333	0.336	0.334	0.024
	**asn**	0.043	0.042	0.045	0.045	0.046	0.043	0.046	0.046	0.046	0.046	0.044	0.048	0.087
	**asp**	0.099	0.099	0.102	0.101	0.100	0.097	0.098	0.101	0.102	0.102	0.100	0.099	0.921
	**β-ala**	0.034	0.033	0.033	0.033	0.034	0.034	0.033	0.033	0.033	0.034	0.034	0.033	0.921
	**glu**	0.113	0.113	0.107	0.107	0.112	0.107	0.108	0.113	0.106	0.113	0.112	0.108	0.921
	**gln**	0.028	0.028	0.027	0.030	0.027	0.030	0.028	0.030	0.024	0.027	0.027	0.025	0.650
	**GSH**	0.029	0.028	0.030	0.029	0.030	0.030	0.029	0.030	0.030	0.030	0.031	0.030	0.650
	**orn**	0.050	0.045	0.056	0.049	0.044	0.046	0.044	0.059	0.060	0.050	0.054	0.048	0.650
	**pro**	0.068	0.067	0.063	0.065	0.067	0.069	0.066	0.067	0.064	0.066	0.068	0.071	0.921
	**put**	0.048	0.048	0.048	0.048	0.048	0.048	0.048	0.048	0.048	0.048	0.048	0.047	0.921
	**ser**	0.047	0.049	0.046	0.047	0.043	0.049	0.047	0.045	0.046	0.049	0.048	0.047	0.921
	**spd**	0.045	0.045	0.045	0.045	0.044	0.044	0.045	0.043	0.042	0.045	0.046	0.047	0.921
**IC**														
	**BIO_SAMPLE_ID**	115	116	117	118	119	120	109	110	111	112	113	114	
	**ORGANISM**	*H. sapiens*	*H. sapiens*	*H. sapiens*	*H. sapiens*	*H. sapiens*	*H. sapiens*	*H. sapiens*	*H. sapiens*	*H. sapiens*	*H. sapiens*	*H. sapiens*	*H. sapiens*	
	**TISSUE**	Calu-3	Calu-3	Calu-3	Calu-3	Calu-3	Calu-3	Calu-3	Calu-3	Calu-3	Calu-3	Calu-3	Calu-3	Calu-3
	**TREATMENT**	mock	mock	mock	mock	mock	mock	CoV-2	CoV-2	CoV-2	CoV-2	CoV-2	CoV-2	mock vs CoV-2
	**TRACER**	13C glc	13C glc	13C glc	13C glc	13C glc	13C glc	13C glc	13C glc	13C glc	13C glc	13C glc	13C glc	p-values
	**average cell number (xE6)**	1.33	1.33	1.33	1.33	1.33	1.33	1	1	1	1	1	1	
	**BIO_REPLICATE**	1	2	3	4	5	6	1	2	3	4	5	6	
	**1,3-BPG**	0.423	0.417	0.414	0.425	0.430	0.427	0.430	0.434	0.430	0.435	0.428	0.423	0.089

	**3-PGA**	0.419	0.417	0.418	0.420	0.417	0.421	0.426	0.428	0.426	0.429	0.430	0.428	<0.001
	**ADP**	0.055	0.056	0.053	0.053	0.056	0.056	0.052	0.054	0.052	0.050	0.053	0.055	0.065
	**α-KG**	0.088	0.085	0.086	0.088	0.090	0.089	0.084	0.087	0.083	0.087	0.085	0.090	0.291
	**AMP**	0.041	0.042	0.038	0.040	0.042	0.041	0.039	0.042	0.038	0.039	0.040	0.041	0.514
	**ATP**	0.045	0.046	0.044	0.046	0.048	0.045	0.046	0.047	0.043	0.045	0.048	0.045	0.870
	**cit**	0.147	0.145	0.146	0.145	0.147	0.142	0.144	0.148	0.141	0.146	0.145	0.149	0.886
	**F-1-P**	0.425	0.429	0.449	0.440	0.444	0.431	0.442	0.446	0.445	0.441	0.458	0.441	<0.001
	**F-1,6-BP**	0.433	0.431	0.434	0.434	0.436	0.434	0.442	0.444	0.443	0.443	0.444	0.445	0.156
	**F-6-P**	0.421	0.439	0.425	0.414	0.424	0.440	0.434	0.429	0.438	0.434	0.442	0.425	0.291
	**fum**	0.110	0.112	0.113	0.112	0.117	0.108	0.107	0.109	0.101	0.107	0.105	0.118	0.223
	**IMP**	0.074	0.077	0.076	0.073	0.078	0.078	0.072	0.073	0.070	0.069	0.068	0.077	0.058
	**icit**	0.251	0.243	0.239	0.258	0.227	0.216	0.212	0.215	0.227	0.224	0.250	0.270	0.689
	**lac**	0.408	0.355	0.428	0.396	0.341	0.371	0.337	0.436	0.437	0.411	0.424	0.358	0.588
	**mal**	0.095	0.093	0.095	0.091	0.096	0.092	0.090	0.092	0.088	0.093	0.092	0.095	0.269
	**PEP**	0.427	0.418	0.420	0.416	0.429	0.000	0.428	0.426	0.420	0.422	0.419	0.379	0.475
	**pyr**	0.369	0.340	0.376	0.316	0.335	0.342	0.329	0.368	0.375	0.355	0.347	0.292	0.886
	**S-7-P**	0.268	0.285	0.270	0.279	0.293	0.286	0.287	0.295	0.290	0.295	0.303	0.297	0.056
	**suc**	0.088	0.087	0.086	0.083	0.085	0.082	0.078	0.083	0.079	0.075	0.082	0.074	0.023
	**UDP**	0.185	0.185	0.180	0.180	0.182	0.187	0.167	0.165	0.162	0.162	0.167	0.170	<0.001
	**UMP**	0.122	0.121	0.116	0.117	0.124	0.124	0.108	0.107	0.103	0.102	0.109	0.109	<0.001
	**UTP**	0.144	0.145	0.139	0.140	0.142	0.146	0.131	0.132	0.124	0.123	0.129	0.133	0.001

Supplementary Table 4. Isotope incorporation ratios (CoV-2/mock for enriched fraction) for amine (Bz)- and anion (IC)-containing metabolites in SARS-CoV-2-infected Calu-3 cells. Metabolite abbreviations are summarized in **Supplementary Table 10**.

ORGANISM	TISSUE	TREATMENT	COMPOUND_ID	Compound/ Pathway	Analysis	n	mean (% 13C labeling)	sd	ratio (CoV-2/mock)	FDR corrected p-value
H. sapiens	**Calu-3**	**CoV-2**	**ala**	**amino acids**	**Bz**	**6**	0.33	0.00855	1.03	2.42E-02
H. sapiens	**Calu-3**	**CoV-2**	**asn**	**amino acids**	**Bz**	**6**	0.05	0.0013	1.25	8.69E-02
H. sapiens	**Calu-3**	**CoV-2**	**asp**	**amino acids**	**Bz**	**6**	0.1	0.00171	1	9.21E-01
H. sapiens	**Calu-3**	**CoV-2**	**β-ala**	**amino acids**	**Bz**	**6**	0.03	0.00028	1	9.21E-01
H. sapiens	**Calu-3**	**CoV-2**	**glu**	**amino acids**	**Bz**	**6**	0.11	0.00303	1	9.21E-01
H. sapiens	**Calu-3**	**CoV-2**	**gln**	**amino acids**	**Bz**	**6**	0.03	0.00213	1	6.50E-01
H. sapiens	**Calu-3**	**CoV-2**	**GSH**	**amino acids**	**Bz**	**6**	0.03	0.00064	1	6.50E-01
H. sapiens	**Calu-3**	**CoV-2**	**orn**	**amino acids**	**Bz**	**6**	0.05	0.00641	1	6.50E-01
H. sapiens	**Calu-3**	**CoV-2**	**pro**	**amino acids**	**Bz**	**6**	0.07	0.00242	1	9.21E-01
H. sapiens	**Calu-3**	**CoV-2**	**put**	**amino acids**	**Bz**	**6**	0.05	0.00047	1	9.21E-01
H. sapiens	**Calu-3**	**CoV-2**	**ser**	**amino acids**	**Bz**	**6**	0.05	0.00148	1	9.21E-01
H. sapiens	**Calu-3**	**CoV-2**	**spm**	**amino acids**	**Bz**	**6**	0.04	0.00151	1	9.21E-01
H. sapiens	**Calu-3**	**CoV-2**	**F-6-P**	**glycolysis**	**IC**	**6**	0.43	0.0061	1.02	2.25E-02
H. sapiens	**Calu-3**	**CoV-2**	**F-1-P**	**glycolysis**	**IC**	**6**	0.45	0.00646	1.02	8.86E-01
H. sapiens	**Calu-3**	**CoV-2**	**1,6-FBP**	**glycolysis**	**IC**	**6**	0.44	0.00077	1.02	2.69E-01

H. sapiens	**Calu-3**	**CoV-2**	**1,3-BPG**	**glycolysis**	**IC**	**6**	0.43	0.00412	1.02	8.92E-02
H. sapiens	**Calu-3**	**CoV-2**	**3-PGA**	**glycolysis**	**IC**	**6**	0.43	0.00169	1.02	6.55E-02
H. sapiens	**Calu-3**	**CoV-2**	**PEP**	**glycolysis**	**IC**	**6**	0.42	0.01812	1.18	2.91E-01
H. sapiens	**Calu-3**	**CoV-2**	**pyr**	**glycolysis**	**IC**	**6**	0.34	0.0302	0.99	5.85E-02
H. sapiens	**Calu-3**	**CoV-2**	**lac**	**glycolysis**	**IC**	**6**	0.4	0.04249	1.05	8.70E-01
H. sapiens	**Calu-3**	**CoV-2**	**cit**	**TCA**	**IC**	**6**	0.15	0.00262	1	6.89E-01
H. sapiens	**Calu-3**	**CoV-2**	**icit**	**TCA**	**IC**	**6**	0.23	0.02222	0.98	2.91E-01
H. sapiens	**Calu-3**	**CoV-2**	**suc**	**TCA**	**IC**	**6**	0.08	0.00372	0.92	ND
H. sapiens	**Calu-3**	**CoV-2**	**α-KG**	**TCA**	**IC**	**6**	0.09	0.00242	0.98	8.86E-01
H. sapiens	**Calu-3**	**CoV-2**	**fum**	**TCA**	**IC**	**6**	0.11	0.00538	0.96	1.12E-04
H. sapiens	**Calu-3**	**CoV-2**	**mal**	**TCA**	**IC**	**6**	0.09	0.00222	0.98	2.10E-05
H. sapiens	**Calu-3**	**CoV-2**	**IMP**	**nucleotides**	**IC**	**6**	0.07	0.00326	0.94	2.10E-05
H. sapiens	**Calu-3**	**CoV-2**	**AMP**	**nucleotides**	**IC**	**6**	0.04	0.00124	0.98	1.56E-01
H. sapiens	**Calu-3**	**CoV-2**	**ADP**	**nucleotides**	**IC**	**6**	0.05	0.00175	0.96	5.14E-01
H. sapiens	**Calu-3**	**CoV-2**	**ATP**	**nucleotides**	**IC**	**6**	0.05	0.00157	1.01	2.23E-01
H. sapiens	**Calu-3**	**CoV-2**	**UMP**	**nucleotides**	**IC**	**6**	0.11	0.00304	0.88	2.49E-05
H. sapiens	**Calu-3**	**CoV-2**	**UDP**	**nucleotides**	**IC**	**6**	0.17	0.00334	0.9	5.55E-02
H. sapiens	**Calu-3**	**CoV-2**	**UTP**	**nucleotides**	**IC**	**6**	0.13	0.00433	0.9	7.66E-04
H. sapiens	**Calu-3**	**CoV-2**	**S-7-P**	**nucleotides**	**IC**	**6**	0.29	0.0056	1.05	5.88E-01

Supplementary Table 5. Data of COVID-19 deceased individuals and control patients for IHC

Patient ID	Gender	Age	SARS-CoV-2	Cause of death and ventilation	Disease Duration (days)	Deceased (category)	lung max viral load (log10/10.000 cells)
**PA-1**	M	77	positive	septic shock, no ventilation, no ECMO	14	early	6.73
**PA-2**	M	81	positive	sepsis, no ECMO	11	early	6.93
**PA-3**	M	62	positive	13d ICU, no ECMO, ARDS, septic shock	19	late	3.81
**PA-4**	F	56	positive	ventilation, ECMO, septic shock, ARDS	27	late	2.46
**PA-5**	M	63	positive	early onset Alzheimer, ventilation 2 days	28	late	2.18
**PA-6**	M	89	positive	ARDS, pneumonia, no ECMO	14	early	5.54
**CO-1**	M	67	NA	MOF cardial, COPD, ECMO	NA	NA	NA
**CO-2**	F	77	NA	intracerebral bleeding; amyloid angiopathy	NA	NA	NA
**CO-3**	M	77	NA	ALS; chronic hypoventilation	NA	NA	NA
**PN-1**	M	59	NA	Pulmonary artery embolism, aspiration pneumonia, dysphagia, cerebral and spinal metastases, unknown primary	NA	NA	NA
**PN-2**	F	94	NA	aspiration pneumonia, lobar pneumonia, dementia	NA	NA	NA
**PN-3**	M	56	NA	septic MOF; pneumonia, polycystic kidney syndrome	NA	NA	NA

Abbreviations: n.a., not applicable; PA, patient/COVID-19; CO, control; PN, patient/pneumonia; ECMO, extracorporeal membrane oxygenation; ICU, intensive care unit; ARDS, acute respiratory distress syndrome; COPD, chronic obstructive pulmonary disease; MOF, multi organ failure; ALS, amyotrophic lateral sclerosis.

Supplementary Table 6. Immunohistochemistry for P62 and LC3 protein in lung samples

Patient ID	Immunohistochemistry counts (4 random fields at 40x)					
	**P62 exp 1**	**LC3 exp 1**	**P62 exp 2**	**LC3 exp 2**	**P62 mean**	**LC3 mean**
**PA-1**	72	16	69	11	70.5	13.5
**PA-2**	98	20	92	16	95.0	18.0
**PA-3**	150	8	140	10	145.0	9.0
**PA-4**	80	38	86	32	83.0	35.0
**PA-5**	85	4	90	7	87.5	5.5
**PA-6**	105	10	119	8	112.0	9.0
**CO-1**	49	11	59	10	54.0	10.5
**CO-2**	30	9	38	12	34.0	10.5
**CO-3**	17	9	25	12	21.0	10.5
**PN-1**	8	12	17	13	12.5	12.5
**PN-2**	86	10	82	8	84.0	9.0
**PN-3**	40	3	46	4	43.0	3.5
**mean patient/COVID-19 (PA)**					98.8	15.0
**mean control (CO)**					36.3	10.5
**mean patient/pneumonia (PN)**					46.5	8.3

Abbreviations: n.a., not applicable; PA, patient/COVID-19; CO, control; PN, patient/pneumonia; ECMO, extracorporeal membrane oxygenation; ICU, intensive care unit; ARDS, acute respiratory distress syndrome; COPD, chronic obstructive pulmonary disease; MOF, multi organ failure; ALS, amyotrophic lateral sclerosis.

Supplementary Table 7. COVID-19 patient and control lung samples analyzed by sNuc-Seq analysis

Patient ID	Gender	Age	SARS-CoV-2	Cause of death and ventilation	Disease Duration (days)	Deceased (category)	Viral load (log10/10,000 cells)	*N* cells	Median genes per cell	Total genes detected	Mean reads per cell	Number of reads
**PA-1**	M	76	positive	septic shock, no ventilation, no ECMO	14	early	6.73	6,412	809	25,849	58,685	376,288,240
**PA-2**	M	81	positive	sepsis, no ECMO	11	early	6.93	3,223	1,219	25,372	97,766	315,100,806
**PA-6**	M	89	positive	ARDS, pneumonia, no ECMO	14	early	5.54	5,915	1,591	26,834	33,702	199,351,673
**PA-7**	M	68	positive	ARDS, acute basal ganglia infarct, ECMO	34	late	1.7	3,506	801	24,547	66,716	233,907,986
**PA-8**	M	72	positive	ARDS, ventilation and ECMO	79	late	2.61	11,582	1,922	27,833	33,245	385,050,204
**PA-9**	M	77	positive	acute liver + renal failure, sepsis, gastric bleeding	84	late	neg	4,727	928	24,756	78,453	370,851,021
**PA-10**	F	68	positive	pulmonary superinfection, septic shock, ventilation and ECMO	34	late	1.2	1,896	868	23,061	118,572	224,812,951
**CO-4**	M	67	NA	MOF, cardial, COPD, ECMO	NA	control	NA	6,724	1,904	27,971	31,182	209,668,599
**CO-5**	F	54	NA	aspiration pneumonia, no ventilation	NA	control	NA	2,840	903	24,369	178,957	508,240,202
**CO-6**	F	94	NA	aspiration pneumonia, lobar pneumonia, dementia, no ventilation	NA	control	NA	4,776	1,302	26,807	157,382	751,660,192

Abbreviations: n.a., not applicable; PA, patient/COVID-19; CO, control; PN, patient/pneumonia; M, male; F, female; ECMO, extracorporeal membrane oxygenation; ARDS, acute respiratory distress syndrome; COPD, chronic obstructive pulmonary disease; MOF, multi organ failure; ALS, amyotrophic lateral sclerosis.

Supplementary Table 8. COVID-19 patients and controls for scSeq analysis of olfactory mucosal samples

Patient ID	Gender	Age	SARS-CoV-2	Clinical outcome	Viral Load	Conc. LOG10 SARS-CoV-2 RNA swab per ml	Days post symptom onset/ PCR test	Days post symptom onset/ scSeq
**PA-11**	M	73	positive	critical	high	5.45	12	13
**PA-12**	M	61	positive	critical	high	5.52	10	11
**PA-13**	M	52	positive	critical	high	7.69	9	10
**PA-14**	M	75	positive	critical	low	4.86	7	7
**PA-15**	M	54	positive	critical	low	4.39	7	7
**PA-16**	M	32	positive	critical	low	3.49	8	8
**PA-17**	M	32	positive	critical	low	3.18	10	11
**PA-18**	M	45	positive	moderate	low	4.40	11	11
**CO-7**	F	34	NA	NA	control	NA	NA	NA
**CO-8**	F	41	NA	NA	control	NA	NA	NA
**CO-9**	M	24	NA	NA	control	NA	NA	NA
**CO-10**	M	33	NA	NA	control	NA	NA	NA
**CO-11**	F	36	NA	NA	control	NA	NA	NA

Abbreviations: n.a., not applicable; PA, patient/COVID-19; CO, control; M, male; F, female

Supplementary Table 9. Overview of compounds

Compound	Concentration Used	Reasoning
AICAR	25 µM	AMPK activation corresponds linearly to AICAR concentration up to 500 µM in isolated hepatocytes[15].
bafilomycin A1	100 nM	100 nM of BafA1 is sufficient to induce maximal LC3B lipidation levels in both cell cultures (**Supplementary Fig. 7**).
DFMO	0.5 mM	0.5 mM is sufficient to inhibit ornithine decarboxylase[16] .
MK-2206	1 µM	0.5 µM is sufficient to inhibit AKT[17].
MRT68921	5 µM	1 µM is sufficient to potently inhibit ULK1 and ULK2[18].
niclosamide	5 µM, 10 µM	10 µM found to stabilize BECN-1[8]. Concentration was reduced to 5 µM when this was determined to be sufficient for antiviral activity.
rapamycin	300 nM	300 nM is sufficient to inhibit mTORC1[19].
SMIP-004	10 µM	10 µM found to stabilize BECN-1[8].
SAR405	1 µM	1 µM is well above the inhibitory concentration for PIK3C3[20].
spermidine	10 µM, 100 µM	100 µM was sufficient to induce autophagy in mammalian cells[21].
spermine	10 µM, 100 µM	100 µM was sufficient to induce autophagy in mammalian cells[21].
valinomycin	5 µM	5 µM found to stabilize Beclin-1[8].

Supplementary Table 10. Overview IC and BZ metabolites

**BZ metabolites**	
AABA	α-aminobutyric acid
adenosine	adenosine
ala	alanine
arg	arginine
asn	asparagine
asp	aspartic acid
carn	carnosine
citru	citrulline
cr	creatine
cys	cysteine
cysgly	cysteinylglycine
cyst	cysteine
GABA	γ-aminobutyric acid
glc	glucose
gln	glutamine
glu	glutamic acid
gly	glycine
GSH	glutathione (reduced)
GSSG	glutathione (oxidized)
hcys	homocysteine
his	histidine
hyp	4-hydroxyproline
ile	isoleucine
leu	leucine
lys	lysine
met	methionine
MTA	5′-methylthioadenosine
NAcput	N-acetylputrescine
NAcspd	N-acetylspermidine
NAcspm	N-acetylspermine
orn	ornithine
phe	phenylalanine
pip. acid	pipecolic acid
pro	proline
put	putrescine
SAH	S-adenosylhomocysteine
SAM	S-adenosylmethionine
ser	serine
spd	spermidine
spm	spermine
tau	taurine
thr	threonine
trp	tryptophan
tyr	tyrosine
val	valine
β-ala	β-alanine
γ-glucys	γ-glutamylcystein
**IC metabolites**	
1,3-BPG	1,3-bisphosphoglyceric acid
3-PGA	3-phosphoglyceric acid
6-PG	6-phosphogluconic acid
aco	aconitic acid
ADP	adenosine diphosphate
AMP	adenosine monophosphate
ATP	adenosine triphosphate
caa	carbamoyl aspartic acid
CDP	cytidine diphosphate
cit	citric acid
CMP	cytidine monophosphate
CTP	cytidine triphosphate
dATP	deoxyadenosine triphosphate
dCTP	deoxycytidine triphosphate
dTMP	deoxythymidine monophosphate
dTTP	deoxythymidine triphosphate
F-1,6-P	fructose-1,6-bisphosphate
F-1-P	fructose-1-phosphate
F-6-P	fructose-6-phosphate
fum	fumaric acid
G-1-P	glucose-1-phosphate
G-3-P	glycerol-3-phosphate

G-6-P	glucose-6-phosphate
GDP	guanosine diphosphate
GMP	guanosine monophosphate
GTP	guanosine triphosphate
icit	isocitric acid
IMP	inosine monophosphate
lac	lactic acid
mal	malic acid
orot. acid	orotic acid
pan	pantothenate
PEP	phosphoenolpyruvic acid
pyr	pyruvic acid
S-7-P	sedoheptulose-7-phosphate
suc	succinic acid
UDP	uridine diphosphate
UDP-Gal	UDP-galactose
UDP-GalNAc	UDP-N-acetylgalactosamine
UDP-Glc	UDP-glucose
UDP-GlcNAc	UDP-N-acetylglucosamine
UMP	uridine monophosphate
UTP	uridine triphosphate
α-KG	α-ketoglutaric acid

Supplementary Table 11. Overview of applied oligonucleotides

Name	Sequence	Source
p62_f	GAAACTGGAGCCCACGTC	in-house
p62_r	TATCCACTT CAATGCCCAGAGG	
p62_p	AATCAGCTGCTGGTCCATCGGAGGA	
hTBP_f	GCTGCGGTAATCATGAGGATAAG	Ref.[22]
hTBP_r	TTCTGGGAAAATGGTGTGCA	
hTBP_p	AGCCACGAACCACGGCACTGATTTT	
E_Sarbeco_F	ACAGGTACGTTAATAGTTAATAGCGT	Ref.[23]
E_Sarbeco_R	ATATTGCAGCAGTACGCACACA	
E_Sarbeco_P1	ACACTAGCCATCCTTACTGCGCTTCG	
ATG5_f	GCTCTTCCTTGGAACATCACAG	in-house
ATG5_r	TCCCATCCAGAGTTGCTTGTG	
ATG5_p	ACCTTCTGCACTGTCCATCTAAGGA	
ATG7_f	TGCCAGCTCGCTTAACATTG	in-house
ATG7_r	AAAGACTCGAGTGTGTTGGTG	
ATG7_p	AGTGCTTTTGACATGAGTGCTCCCA	
BECN1_f	TGAGTGTCAGAACTACAAACGC	in-house
BECN1_r	AGCCTGGACCTTCTCGAG	
BECN1_p	TCTTTTCCACGTCTTCCAGCTCCTG	
FIP200_f	GTGATCGTCCACCTGCTATTC	in-house
FIP200_r	TCTTGGCAACTTCATACATTTCC	
FIP200_p	CAAGCTGTGTCCTTGAAGCAACAGT	

References (SUPPLEMENTARY data)

1 Narayanan, K. *et al.* Severe acute respiratory syndrome coronavirus nsp1 suppresses host gene expression, including that of type I interferon, in infected cells. *J Virol* **82**, 4471-4479, doi:10.1128/JVI.02472-07 (2008).

2 Schubert, K. *et al.* SARS-CoV-2 Nsp1 binds the ribosomal mRNA channel to inhibit translation. *Nat Struct Mol Biol* **27**, 959-966, doi:10.1038/s41594-020-0511-8 (2020).

3 Tanaka, T., Kamitani, W., DeDiego, M. L., Enjuanes, L. & Matsuura, Y. Severe acute respiratory syndrome coronavirus nsp1 facilitates efficient propagation in cells through a specific translational shutoff of host mRNA. *J Virol* **86**, 11128-11137, doi:10.1128/JVI.01700-12 (2012).

4 Thoms, M. *et al.* Structural basis for translational shutdown and immune evasion by the Nsp1 protein of SARS-CoV-2. *Science* **369**, 1249-1255, doi:10.1126/science.abc8665 (2020).

5 Klann, K., Tascher, G. & Munch, C. Functional Translatome Proteomics Reveal Converging and Dose-Dependent Regulation by mTORC1 and eIF2alpha. *Mol Cell* **77**, 913-925 e914, doi:10.1016/j.molcel.2019.11.010 (2020).

6 Angelini, M. M., Akhlaghpour, M., Neuman, B. W. & Buchmeier, M. J. Severe acute respiratory syndrome coronavirus nonstructural proteins 3, 4, and 6 induce double-membrane vesicles. *mBio* **4**, doi:10.1128/mBio.00524-13 (2013).

7 Oudshoorn, D. *et al.* Expression and Cleavage of Middle East Respiratory Syndrome Coronavirus nsp3-4 Polyprotein Induce the Formation of Double-Membrane Vesicles That Mimic Those Associated with Coronaviral RNA Replication. *mBio* **8**, doi:10.1128/mBio.01658-17 (2017).

8 Gassen, N. C. *et al.* SKP2 attenuates autophagy through Beclin1-ubiquitination and its inhibition reduces MERS-Coronavirus infection. *Nature Communications* **10**, 1-16 (2019).

9 Hayn, M. *et al.* Systematic functional analysis of SARS-CoV-2 proteins uncovers viral innate immune antagonists and remaining vulnerabilities. *Cell Rep*, 109126, doi:10.1016/j.celrep.2021.109126 (2021).

10 Zhang, Y. *et al.* The SARS-CoV-2 protein ORF3a inhibits fusion of autophagosomes with lysosomes. *Cell Discov* **7**, 31, doi:10.1038/s41421-021-00268-z (2021).

11 Miao, G. *et al.* ORF3a of the COVID-19 virus SARS-CoV-2 blocks HOPS complex-mediated assembly of the SNARE complex required for autolysosome formation. *Dev Cell* **56**, 427-442 e425, doi:10.1016/j.devcel.2020.12.010 (2021).

12 Schroeder, S. *et al.* Interferon antagonism by SARS-CoV-2: a functional study using reverse genetics. *Lancet Microbe* **2**, e210-e218, doi:10.1016/S2666-5247(21)00027-6 (2021).

13 Cornillez-Ty, C. T., Liao, L., Yates, J. R., 3rd, Kuhn, P. & Buchmeier, M. J. Severe acute respiratory syndrome coronavirus nonstructural protein 2 interacts with a host protein complex involved in mitochondrial biogenesis and intracellular signaling. *J Virol* **83**, 10314-10318, doi:10.1128/JVI.00842-09 (2009).

14 Davies, J. P., Almasy, K. M., McDonald, E. F. & Plate, L. Comparative Multiplexed Interactomics of SARS-CoV-2 and Homologous Coronavirus Nonstructural Proteins Identifies Unique and Shared Host-Cell Dependencies. *ACS Infect Dis* **6**, 3174-3189, doi:10.1021/acsinfecdis.0c00500 (2020).

15 Corton, J. M., Gillespie, J. G., Hawley, S. A. & Hardie, D. G. 5-Aminoimidazole-4-carboxamide ribonucleoside: a specific method for activating AMP-activated protein kinase in intact cells? *European journal of biochemistry* **229**, 558-565 (1995).

16 Mounce, B. C. *et al.* Inhibition of Polyamine Biosynthesis Is a Broad-Spectrum Strategy against RNA Viruses. *J Virol* **90**, 9683-9692, doi:10.1128/JVI.01347-16 (2016).

17 Cheng, Y. *et al.* MK-2206, a novel allosteric inhibitor of Akt, synergizes with gefitinib against malignant glioma via modulating both autophagy and apoptosis. *Molecular cancer therapeutics* **11**, 154-164 (2012).

18 Petherick, K. J. *et al.* Pharmacological inhibition of ULK1 kinase blocks mammalian target of rapamycin (mTOR)-dependent autophagy. *Journal of Biological Chemistry* **290**, 11376-11383 (2015).

19 Guertin, D. A. & Sabatini, D. M. The pharmacology of mTOR inhibition. *Sci. Signal.* **2**, pe24-pe24 (2009).

20 Pasquier, B. SAR405, a PIK3C3/Vps34 inhibitor that prevents autophagy and synergizes with MTOR inhibition in tumor cells. *Autophagy* **11**, 725-726 (2015).
21 Morselli, E. *et al.* Spermidine and resveratrol induce autophagy by distinct pathways converging on the acetylproteome. *J Cell Biol* **192**, 615-629, doi:10.1083/jcb.201008167 (2011).
22 Biesold, S. E. *et al.* Type I interferon reaction to viral infection in interferon-competent, immortalized cell lines from the African fruit bat Eidolon helvum. *PLoS One* **6**, e28131, doi:10.1371/journal.pone.0028131 (2011).
23 Corman, V. M. *et al.* Detection of 2019 novel coronavirus (2019-nCoV) by real-time RT-PCR. *Euro Surveill* **25**, doi:10.2807/1560-7917.ES.2020.25.3.2000045 (2020).
