# Supplementary material for: SARS-CoV-2-mediated dysregulation of metabolism and autophagy uncovers host-targeting antivirals
Source: Nat Commun. 2021 Jun 21;12:3818. doi: 10.1038/s41467-021-24007-w (PMC8217552; doi:10.1038/s41467-021-24007-w)
Supplement: Supplementary file 3 — Reporting Summary [file 41467_2021_24007_MOESM3_ESM.pdf]

nature research

Corresponding author(s): Nils C. Gassen and Marcel A. Müller

Last updated by author(s): May 19, 2021

Reporting Summary

Nature Research wishes to improve the reproducibility of the work that we publish. This form provides structure for consistency and transparency in reporting. For further information on Nature Research policies, see our Editorial Policies and the Editorial Policy Checklist.

Statistics

For all statistical analyses, confirm that the following items are present in the figure legend, table legend, main text, or Methods section.

n/a	Confirmed	
☐	☒	The exact sample size (n) for each experimental group/condition, given as a discrete number and unit of measurement
☐	☒	A statement on whether measurements were taken from distinct samples or whether the same sample was measured repeatedly
☐	☒	The statistical test(s) used AND whether they are one- or two-sided<br>*Only common tests should be described solely by name; describe more complex techniques in the Methods section.*
☒	☐	A description of all covariates tested
☐	☒	A description of any assumptions or corrections, such as tests of normality and adjustment for multiple comparisons
☐	☒	A full description of the statistical parameters including central tendency (e.g. means) or other basic estimates (e.g. regression coefficient) AND variation (e.g. standard deviation) or associated estimates of uncertainty (e.g. confidence intervals)
☐	☒	For null hypothesis testing, the test statistic (e.g. F, t, r) with confidence intervals, effect sizes, degrees of freedom and P value noted<br>*Give P values as exact values whenever suitable.*
☒	☐	For Bayesian analysis, information on the choice of priors and Markov chain Monte Carlo settings
☒	☐	For hierarchical and complex designs, identification of the appropriate level for tests and full reporting of outcomes
☒	☐	Estimates of effect sizes (e.g. Cohen's d, Pearson's r), indicating how they were calculated

Our web collection on statistics for biologists contains articles on many of the points above.

Software and code

Policy information about availability of computer code

Data collection	LC-MS data was collected using the Chromeleon 7.2 software suite (Thermo Fisher Scientific) or MassLynx 4.1 (Waters). Targeted compound peak integration was done using Tracfinder 4.2 (Thermo Fisher Scientific) or TargetLynx (Waters).
Data analysis	Data analysis was performed using MetaboAnalyst 4.0<br>AxioVision software ZEN Pro 2<br>Seurat 3.1.4<br>Cell ranger 3.0.1<br>10x Genomics, version 3.1.0<br>TraceFinder software (Version 4.1)<br>InstantClue (v.0.10.10.dev-snap)<br>SIMCA 13 (Umetrics)

For manuscripts utilizing custom algorithms or software that are central to the research but not yet described in published literature, software must be made available to editors and reviewers. We strongly encourage code deposition in a community repository (e.g. GitHub). See the Nature Research guidelines for submitting code & software for further information.

Data

Policy information about availability of data

All manuscripts must include a data availability statement. This statement should provide the following information, where applicable:

- Accession codes, unique identifiers, or web links for publicly available datasets
- A list of figures that have associated raw data
- A description of any restrictions on data availability

Metabolomics data shown in Fig. 1 and 5, Extended Data Fig. 1-4, 12-13, Extended Data Tables 1-4) are available at the Metabolomics data repository MetaboLights under the study identifier MTBLS2840. Histopathological lung datasets generated and analyzed during the current study (Fig. 3b) are available on reasonable request. The sequencing data of Fig. 3c, Extended Data Fig. 9 are available under controlled access and require a Data Transfer Agreement in the European Genome-pheome Archive repository: (EGAS00001004689). RNAseq data mentioned in Extended Data Fig. 5 and 8 are deposited: GSE148729, GSE147507, and GSE162208. Source data are provided with this paper.
RNAseq data was used from Wyler et al 2021 iScience https://doi.org/10.1016/j.isci.2021.102151

Field-specific reporting

Please select the one below that is the best fit for your research. If you are not sure, read the appropriate sections before making your selection.

☒ Life sciences ☐ Behavioural & social sciences ☐ Ecological, evolutionary & environmental sciences

For a reference copy of the document with all sections, see nature.com/documents/nr-reporting-summary-flat.pdf

Life sciences study design

All studies must disclose on these points even when the disclosure is negative.

Sample size	No calculations were performed to pre-determine sample size. Where applicable, experiments were performed under the hypothesis that potential effect sizes would approximate those of previously examined MERS-CoV, for which three biological replicates were sufficient to characterize significant differences (Gassen et al., Nat. Comm. 2019). When individual cells were analysed for counting autophagosomes and autolysosomes, at least 10 cells were analysed, again based on previous experience (Gassen et al., Nat. Comm. 2019) and in accordance with established guidelines for monitoring autophagy (Kilonsky et al. Autophagy. 2021). As the effect size of the metabolomics experiments was unknown prior to this work, we pre-determined they should be performed in biological quadruplicate, to enhance sensitivity to potentially small effect sizes and better ensure that the results would be suitable for subsequent multivariate analysis (Worley and Powers, Curr. Metabolomics. 2013). Our hamster data reflect the sample sizes from the study in which they originated (Osterrieder et al. Viruses. 2020) and the design of ex vivo experiments was contingent on limited availability of postmortem COVID-19 tissue (Elezkurtaj et al., Sci. Rep. 2021).
Data exclusions	C13 VeroFM metabolomics data were excluded as there were technical or experimental issues with this dataset that we lack the capacity to fully contextualize via further experiments in a timely manner. All experiments were done with at least n = 3 biologically independent samples. Removal of single outliers due to technical issues are mentioned in the respective Figure legends.
Replication	The cell experiments were performed in at least three biological replicates with exception of Fig. 4. n = 2 for spd, spm, AICAR. Metabolomics experiments were perfomed with four biological replicates. All attempts at replication were successful.
Randomization	Each cell culture experiment involved seeding plates of resuspended cells from the same source. Plates were examined by microscopy prior to experimentation to ensure a consistent growth phenotype and arbitrarily assigned to experimental and control groups. Organoids were likewise controlled. Hamster groups were selected as descibed previously (Osterrieder et al. Viruses. 2020). Postmortem lung tissue was randomly sourced, according to inclusion criteria, from an autopsy cohort (Elezkurtaj et al., Sci. Rep. 2021).
Blinding	The counting of autolysosomes and autophagosomes was performed by a colleague blind to the experimental conditions. For all other experiments, samples were pseudoanonymized before analysis (e.g. by RT-PCR or virus plaque titrations or Westernblot anaysis).

Reporting for specific materials, systems and methods

We require information from authors about some types of materials, experimental systems and methods used in many studies. Here, indicate whether each material, system or method listed is relevant to your study. If you are not sure if a list item applies to your research, read the appropriate section before selecting a response.

Materials & experimental systems

n/a	Involved in the study
☐	☒ Antibodies
☐	☒ Eukaryotic cell lines
☒	☐ Palaeontology and archaeology
☐	☒ Animals and other organisms
☐	☒ Human research participants
☒	☐ Clinical data
☒	☐ Dual use research of concern

Methods

n/a	Involved in the study
☒	☐ ChIP-seq
☒	☐ Flow cytometry
☒	☐ MRI-based neuroimaging

Antibodies

Antibodies used	The following primary antibodies were used: β-actin (1:5,000 Cell Signalling Technology, #8457), SQSTM1/p62 (1:1,000, Cell Signalling Technology, #5114), LC3B (1:1,000, Cell Signalling Technology, #3868), BECN1 (1:1,000, Cell Signalling Technology, #3738), pBECN1S15 (1:1,000, Cell Signalling Technology, #84966), ATG14 (1:1,000, Cell Signalling Technology, #5504), pATG14S29 (1:1,000, Cell Signalling Technology, #13155), ULK1 (1:1,000, Cell Signalling Technology, #8054), pULK1 (S555) (1:1,000, Cell Signalling Technology, #5869), pULK1 (S757) (1:1,000, Cell Signalling Technology, #6888), TSC2 (1:1,000, Cell Signalling Technology, #3612), pTSC2 (S1387) (1:1,000, Cell Signalling Technology, #5584), AMPKα (1:1,000, Cell Signalling Technology, #2532), pAMPK (T172) (1:1,000, Cell Signalling Technology, #2531), pAMPK substrate motif (1:1,000, Cell Signalling Technology, #5759), pAKT (S473) (1:1,000, Cell Signalling Technology, #4060), AKT (1:1,000, Cell Signalling Technology, #9272), HSC70 (1:5,000, Enzo Life Sciences, ADI-SPA-757-F), SKP2 (1:1,000, Cell Signalling Technology, #2652), pSKP2 (S64) (1:1,000, Cell Signalling Technology, #14865). Anti-pSKP2 (S72) was a kind gift by Cell Signalling Technology (used 1:1,000). Undiluted hybridoma culture supernatant of an in-house mouse monoclonal anti-SARS-CoV-2 [illegible]ibody (#G229FA10; kindly provided by J. A. Schenk and F. Sellrie; UP Transfer GmbH, Potsdam). Secondary antibodies: an[illegible]bit IgG, HRP-linked antibody (1:10,000, Cell Signalling Technology, #7074), anti-mouse IgG, HRP-linked antibody (1:10,000, Cell Signalling Technology, #7076) and Cy3-labeled polyclonal goat-anti mouse antibody (1:200, Dianova, 115-165-166). Subsequently, blots were washed and probed with the respective horseradish peroxidase- (or fluorophore-conjugated) secondary antibody for 1 h at room temperature.
Validation	The monoclonal SARS-CoV-2 anti-Spike antibody-containing hybridoma culture supernatant #G229FA10 was validated for WB in-house using recombinantly expressed Spike protein (Institute of Virology, Charité). All others were validated by the manufacturer. Primary antibodies: β-actin #8457 (validated for WB, IF, F; species reactivity H, M, R, Mk, Dm, Z); SQSTM1/p62 #5114 (validated for WB; species reactivity H, M, R, Mk); LC3B #3868 (validated for WB, IF, F; species reactivity H); BECN1 #3738 (validated for WB, IP; species reactivity H, M, R); pBECN1S15 #84966 (validated for WB; species reactivity H, M); ATG14 #5504 (validated for WB; species reactivity H); pATG14S29 #13155 (validated for WB; species reactivity H, M); ULK1 #8054 (validated for WB, IP; species reactivity H, M, R, Mk); pULK1 (S555) #5869 (validated for WB, IP; species reactivity H, M); pULK1 (S757) #6888 (validated for WB; species reactivity H, M, Mk); TSC2 #3612 (validated for WB; species reactivity H, M, R, Mk); pTSC2 (S1387) #5584 (validated for WB, IP; species reactivity H, M, R, Mk); AMPKα #2532 (validated for WB, IP; species reactivity H, M, R, Hm, Mk); pAMPK (T172) #2531 (validated for WB; species reactivity H, M, R, Mk); pAMPK substrate motif #5759 (validated for WB, IP; species reactivity all); pAKT (S473) #4060 (validated for WB, IP, IHC, IF,F; species reactivity H, M, R, Hm, Mk, Dm, Z, B); AKT #9272 (validated for WB, IP, IF, F; species reactivity H, M, R, Hm, Mk, C, Dm, B, Dg, Pg, GP); HSC70 ADI-SPA-757-F (validated for WB, IHC; species reactivity H, M, R, B, Dg, Dm, Mk, Pg, GP, R, C, Sh, Sc, Pt); SKP2 #2652 (validated for WB, IP, IHC, IF; species reactivity H, Mk); pSKP2 (S64) #14865 (validated for WB, IP; species reactivity H), pSKP2 (S72) (validated for WB; species reactivity H). Secondary antibodies were also evaluated for cross-reactivity by the manufacturer and used consistently with the manufacturer's recommendations.

Eukaryotic cell lines

Policy information about cell lines

Cell line source(s)	VeroFM (ATCC CCL-81), VeroE6 (ATCC CRL-1586), NCI-H1299 (ATCC CRL-5803); Calu-3 (ATCC HTB-55)
Authentication	Upon purchase by cytochrome b PCR.
Mycoplasma contamination	All applied cells were tested negative for mycoplasma (PCR-based methods).
Commonly misidentified lines (See ICLAC register)	No commonly misidentified cell lines were used in the study.

Animals and other organisms

Policy information about studies involving animals; ARRIVE guidelines recommended for reporting animal research

Laboratory animals	Syrian hamsters (Mesocricetus auratus)
Wild animals	NA
Field-collected samples	NA

Ethics oversight	The animal use protocol was approved by the Landesamt für Gesundheit und Soziales in Berlin, Germany (approval number 0086/20; approved on 30.04.2020) to co-author Jakob Trimpert.

Note that full information on the approval of the study protocol must also be provided in the manuscript.

Human research participants

Policy information about studies involving human research participants

Population characteristics	NA
Recruitment	Patients were recruited upon admission to Charité.
Ethics oversight	The use of primary lung tissue was approved by the Charité ethics committee (projects EA2/079/13 to co-author Andreas Hocke) and written informed consent was obtained from all patients. The use of COVID-19 patient samples applied in histopathology and sequencing was approved by the Ethics Committee of the Charité (EA1/144/13, EA2/066/20 and EA1/075/19 to co-author Helena Radbruch) as well as by the Charité-BIH COVID-19 research board and complied with the Declaration of Helsinki. The generation and cultivation of human primary intestinal organoid cultures was done under the ethics approval no. EA4/164/19 (to co-author Markus Morkel).

Note that full information on the approval of the study protocol must also be provided in the manuscript.
